# Surface Adsorption and Photoinduced Degradation: A Study of Spinel Ferrite Nanomaterials for Removal of a Model Organic Pollutant from Water

**DOI:** 10.1021/acs.chemmater.3c01986

**Published:** 2024-04-24

**Authors:** Karla R. Sanchez-Lievanos, Tong Sun, Elise A. Gendrich, Kathryn E. Knowles

**Affiliations:** Department of Chemistry, University of Rochester, Rochester, New York 14627, United States

## Abstract

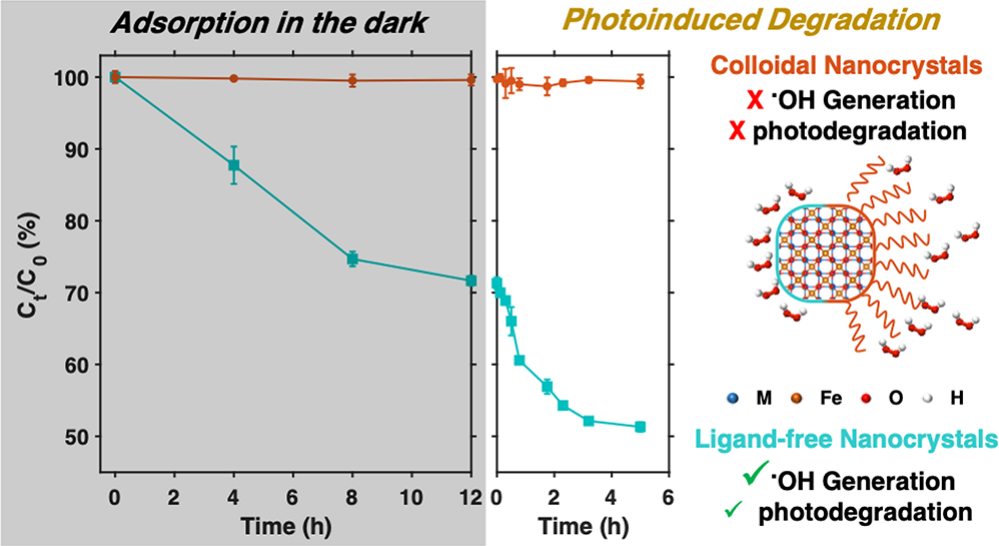

Spinel oxide nanocrystals are attractive materials for photoinduced advanced oxidation processes that degrade organic pollutants in water due to their chemical stability and tunability, visible light absorption, and magnetic recoverability. However, a systematic understanding of the structural and chemical factors that control the reactivity of specific spinel oxide nanocrystal materials toward photoinduced degradation processes is lacking. This Perspective illustrates these knowledge gaps through an investigation into the impacts of surface chemistry and composition of spinel ferrite nanocrystals of formula MFe_2_O_4_ (M = Mg, Fe, Co, Ni, Cu, Zn) on their ability to remove a model organic pollutant (methyl orange (MO)) from water. We identify two mechanisms by which the nanocrystals remove MO from water: (*i*) surface adsorption and (*ii*) photoinduced degradation under visible light irradiation in the presence of hydrogen peroxide via the photo-Fenton reaction. Nanocrystals that do not contain any surface ligands are more effective at removing MO from water than nanocrystals that contain surface ligands, despite our observation that the ligand-less nanocrystals do not form stable colloidal dispersions in water, while ligand-coated nanocrystals are colloidally stable. For many of the spinel ferrite compositions studied here, the fraction of methyl orange removal via adsorption to the nanocrystal surface in the absence of photoexcitation is larger than the fraction removed under irradiation. Our data indicate that the composition-dependent surface charge of the nanocrystals controls the degree of surface adsorption of the charged MO molecule. Overall, these results demonstrate that careful consideration of the impacts of surface chemistry on the behavior of spinel ferrite nanocrystals is required to accurately assess and subsequently understand their activity toward the photoinduced degradation of organic molecules.

## Introduction

**Figure 1:**
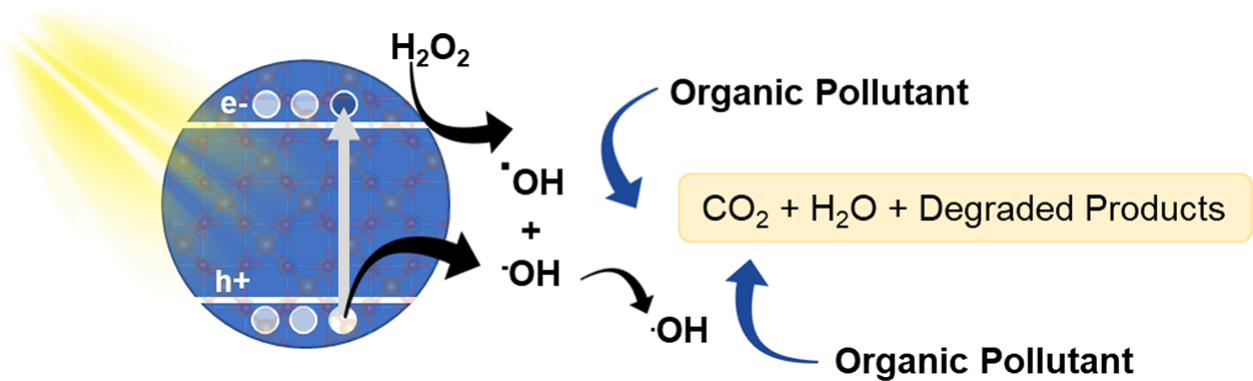
Scheme depicting the general mechanism of photoinduced oxidative degradation of organic pollutants in the presence of a semiconductor material, hydrogen peroxide, and a light source.

The “zero” waste strategy in the water and wastewater treatment industries envisions the application of effective and sustainable oxidation processes, including solar-driven photodegradation processes, to remove contaminants.^1^ Sustainable advanced oxidation processes (AOPs) are technologies that produce highly reactive oxygen species (ROS) in situ. For example, a common approach is to irradiate a photoactive material in the presence of H_2_O_2_ to produce hydroxyl radicals (HO^•^) that are capable of oxidizing virtually any organic compound present in a water matrix (Figure 1).^2^ Many of the studied systems involve photogenerated Fe^2+^ species as the reducing agent that converts H_2_O_2_ to HO^•^ and HO^–^.^3^ This reaction is known as the photo-Fenton process after the Fenton reaction between Fe^2+^ ions and H_2_O_2_. In general, photogeneration of HO^•^ radicals from H_2_O_2_ is often termed a “photo-Fenton-like” process.^3^ These processes possess a smaller footprint compared to conventional oxidation processes, like electrocoagulation and aerobic biological treatments, that often require high capital and operating costs and are not effective in fully degrading the contaminants.^4^ In photoinduced contaminant removal processes, the quantity and redox capacity of photogenerated electron–hole pairs are primarily responsible for the contaminant degradation and determine the efficiency of the desired photoassisted reaction.^5^ To date, some of the most widely investigated photodriven systems for contaminant removal comprise wide-band-gap metal oxide semiconductors, such as TiO_2_,^6,7^ Ga_2_O_3_,^8,9^ and In_2_O_3_,^10,11^ that are active only under ultraviolet (UV) irradiation. Although UV light contains more energy per photon than visible light, UV light only represents ∼6% of the solar spectrum, whereas visible light makes up to ∼27% of the total available number of photons from incoming solar radiation. Moreover, visible light has longer penetration depths in water than ultraviolet light.^12,13^ To benefit from a greater amount of energy available from the sun, photoactive materials that can use both UV and visible light are preferred over those that use UV light alone. In this context, major efforts have been made over the past few years to enhance the ability of visible-light-absorbing semiconductor materials to facilitate light-induced reactions, notably by modifying their properties at the nanoscale.^14,15^

The search for optimal semiconductors to mediate these photoinduced oxidative degradation processes has identified spinel ferrite nanocrystals (MFe_2_O_4_) as promising candidates due to their exceptional chemical stability and potential magnetic recoverability.^16,17^ Although there are dozens of studies reporting on the reactivity of spinel ferrites toward the photo-Fenton degradation reaction, there is currently a lack of a systematic understanding of the specific factors that limit the performance of these materials. In general, there are two primary factors that impact potential photoreactivity of semiconductor materials at the nanoscale: (*i*) band-edge redox potentials and (*ii*) surface chemistry. The band-edge redox potentials define the limits of the thermodynamic driving force for a targeted redox process.^18^ Although wide-band-gap materials exhibit deficient light absorption, having a large band gap enhances their redox driving force. In contrast, materials with smaller band gaps can absorb a wider range of wavelengths from the solar spectrum but possess a more limited redox driving force. Unlike colloidal quantum dots, where band-edge potentials can be tuned by changing nanocrystal size through quantum confinement, many transition metal oxides rely on composition tuning or doping to induce such changes in their band-edge potentials.^19,20^ Additionally, changes in the coverage and identity of ligands bound to the nanocrystal surface can either enhance or inhibit the ability of substrates to approach and/or adsorb to the nanocrystal surface.^21^ Close proximity between substrates and the nanocrystal surface is required to achieve efficient transfer of photogenerated charge carriers, which is a crucial step in the photoreaction process. Mid-gap states associated with surface defects can further constrain the driving force for photoinduced redox processes, but these states may also promote the surface localization of charges and therefore help “shepherd” photogenerated charges to surface-bound substrates.

Comparative assessment of the performance of various spinel ferrite nanocrystals in the photo-Fenton reaction has been hampered by the lack of well-defined standard conditions under which to quantify the activity of these materials. Furthermore, many reports, including this one, use nanomaterials that do not form stable colloidal suspensions of high optical quality but rather exhibit significant scattering that makes accurate quantification of quantum efficiency practically impossible. Table 1 summarizes results from recent studies of the photoinduced degradation of various emergent organic pollutants in the presence of spinel ferrite nanocrystals. For studies that examined multiple spinel ferrite materials, we include in Table 1 the material that exhibited the most efficient degradation performance. Most of these reactions were allowed to reach adsorption–desorption equilibrium between the dye solution and the nanocrystals in the dark after 30 min to 1 h under constant stirring. However, these studies vary in several key variables, such as nanocrystal load, pollutant concentration, presence and identity of surface ligands, incident photon flux, and duration of experiment. Attempting to find a significant parameter with which to compare their performance with the available information, we transformed the nanocrystal load to mol % nanocrystals (assuming the reported mass used of each nanocrystal corresponds to pure MFe_2_O_4_) to compare the molar amounts of used nanocrystals to the molar concentration of pollutant to be degraded. In general, as noted in Table 1, superstoichiometric amounts of MFe_2_O_4_ are often employed in these types of reactions. We note that even if the molar amount of MFe_2_O_4_ used is in the range of 1–3 orders of magnitude higher than the number of moles of pollutant (mol % of 1000–100000), the concentration of surface sites available to adsorb substrates and participate in the targeted reactions is much smaller and may even be stoichiometric or substoichiometric. However, methods to estimate the relevant concentration of surface sites are susceptible to large uncertainties due to the highly heterogeneous nature of nanostructured MFe_2_O_4_ materials and their tendency to form aggregates in solution. For example, Gao et al. examined the ability of hierarchical spherical aggregates of ZnFe_2_O_4_ nanoparticles to mediate the photoinduced degradation of Terramycin, also known as oxytetracycline, which is an emerging antibiotic pollutant.^22^ The primary particles have a diameter of roughly 5 nm, and the spherical aggregates have an average diameter of 210 nm. Accounting for a ZnFe_2_O_4_ lattice parameter of 0.842 nm and a surface atomic layer formed by Fe^3+^ and Zn^2+^ cations of approximately 0.138 nm thickness, about 16% of the total metal ions are on the surface of a 5 nm particle, whereas only 0.4% of the total metal ions are on the surface of a 210 nm particle (note that this estimate does not account for the surface roughness of the aggregate). Taking the surface percentage of the 210 nm aggregates to be the relevant value, the surface ions on these aggregates ultimately account for ∼10 μM in the 0.6 g/L (2.5 mM) bulk ZnFe_2_O_4_ load. Hence, in this reaction, there is around 11 mol % active nanocrystal surface sites relative to the pollutant that when compared to the original bulk 2900 mol % loading (see Table 1) indicates a concentration that is much closer to substoichiometric (see Table S1 in the Supporting Information for detailed calculations). We note that this approximation of the concentration of surface sites is subject to a very large uncertainty arising from the polydispersity of the spherical aggregates (reported to be ∼10%) and heterogeneity in their surface roughness.^22^

**Table 1:** Summary of Recent Reports of Spinel Ferrites Used in the Photoinduced Degradation of Emerging Pollutants in Water^a^

material	pollutant	pollutant conc (mM)	light source	power (W)	surface ligands	MFe_2_O_4_ load (mM)	mol% MFe_2_O_4_	pH	*t* (min)	deg (%)	ref
CoFe_2_O_4_^b^^,^^c^	methylene blue	0.031	UV-C lamp	32^d^		2.1	6800	7	5	93	(23)
CoFe_2_O_4_	methylene blue	0.001	Xe lamp/UV cutoff filter	300^e^		4.3	430000		420	92	(24)
CoFe_2_O_4_^b^	methylene blue	0.031	solar simulator			0.043	140		140	80	(25)
CoFe_2_O_4_	Alizarin red S	0.028	UV/vis		APTES	0.043	150	7	180	91	(26)
CoFe_2_O_4_	Rhodamine 6G	0.01	UV Hg lamp	15^d^		0.43	4100	9	240	91	(27)
ZnFe_2_O_4_	4-chlorophenol	0.23	UV	2^d^	oxalic acid^f^	3.1	1300	3	180	69	(28)
ZnFe_2_O_4_	diclofenac sodium salt	0.06	UV	30^d^		4.1	6600		120	61	(29)
ZnFe_2_O_4_^b^	oxy-tetracycline	0.09	Xe lamp	300^e^	polyacrylic acid^f^	2.5	2900	7	60	97	(22)
Cu_0.8_Mn_0.2_Fe_2_O_4_^b^	tetracycline hydrochloride	0.17	Xe lamp/UV cutoff filter	300^e^		0.42	250	11	30	94	(30)
NiFe_2_O_4_	methylene blue	0.006	Xe lamp	150^e^		4.3	68000		150	79	(31)
Co_0.5_Zn_0.5_Fe_2_O_4_^b^	methylene blue	0.031	W lamp	300^e^		0.21	670		60	77	(32)
MgFe_2_O_4_	methylene blue	0.031	Xe lamp/420 nm cutoff filter	150^e^	Me_4_NOH^f^	5	16000		360	26	(33)
MgFe_2_O_4_	methylene blue	0.016	UV lamp at 366 nm			1.3	8000		300	16	(34)

a Abbreviations: conc: concentration; deg: degradation.

b H_2_O_2_ is used in the reaction system.

c No dark equilibration period.

d Optical power.

e Electric power.

f Annealed.

Further analysis of the data compiled in Table 1 reveals some apparent discrepancies in the reported pollutant removal efficiency among reports that examined the same combination of metal ferrite and pollutant molecule. For example, various reports have investigated the potential activity of CoFe_2_O_4_ nanostructured materials for the photoinduced degradation of methylene blue. Research from Gupta et al.,^23^ Tavana et al.,^24^ and Kalam et al.^25^ reported removal efficiencies for this nanocrystal/pollutant combination of 93, 92, and 80%, respectively. Yet, given the inherent variations among their reaction systems, it is very difficult to compare these values. Tavana et al. utilized a xenon lamp with a UV cutoff filter and 300 W of electrical power with a catalyst dose of 1 g/L in a 0.3 mg/L methylene blue solution. The reported degradation of 92% of the dye occurs over 7 h.^24^ On the other hand, Gupta et al. employed a UV-C lamp with 32 W of optical power coupled to 5 mM of H_2_O_2_ and 0.5 g/L of catalyst load, leading to degradation of 93% of the methylene blue in a solution with an initial concentration of 10 mg/L in only 5 min.^23^ Alternatively, Kalam et al. used one of the lowest nanocrystal loads reported in the literature for this type of material, 0.01 g/L. Their experimental conditions include a solar simulator and 5 mM of H_2_O_2_ that degrade 80% of a 10 mg/L methylene blue solution in 140 min.^25^ Ultimately, the CoFe_2_O_4_ nanomaterials used in these studies, although similar in overall stoichiometry, present different morphologies, surface chemistries, and crystallite sizes, which may explain some of the disparities in their reported activities. However, many of the differences in these reported degradation efficiencies may also arise from differences in the incident optical power and the concentrations of H_2_O_2_, nanocrystals, and organic pollutant.

In an effort to uncover the factors inherent to the material, rather than the experimental setup, that drive activity toward photoinduced degradation, we present here a systematic study of the ability of a series of metal ferrite nanocrystals to remove a model pollutant, the azo dye methyl orange, from water via the photo-Fenton reaction under identical illumination conditions and similar nanocrystal loadings. We chose methyl orange as a model compound for several reasons: (*i*) It is a synthetic dye that imparts a distinct color to the water, making it easy to visually monitor the degradation process and track the efficiency through UV–vis spectroscopy. (*ii*) Synthetic dye molecules like methyl orange account for a significant percentage of water contamination, and azo dyes represent over 50% of all industrial dyes.^35^ With its aromatic rings and azo group, methyl orange therefore possesses a chemical structure that is representative of many organic pollutants found in water. (*iii*) Methyl orange is relatively inexpensive compared to some other organic pollutants. Using it as a model pollutant allows for cost-effective experimentation and testing of the photocatalytic degradation methods.

In contrast to previous studies, we scrutinize the impact of surface chemistry as well as the bulk composition of ferrite on photo-Fenton reactivity. Since the optimization and control of nanocrystal surface chemistry can be modulated by surface ligands, here we present an adapted ligand exchange protocol to promote surface functionalization with four types of water solubilizing ligands, namely, citric acid (CA), mercaptosuccinic acid (MSA), nitrodopamine (NDA), and (aminomethyl)phosphonic acid (AMPA). We use NiFe_2_O_4_ as a model system to investigate the impact of surface functionalization on the ability of spinel ferrite nanocrystals to generate hydroxyl radicals (HO^•^) under illumination in the presence of H_2_O_2_. We find that the presence of any kind of surface ligand limits the ability of NiFe_2_O_4_ nanocrystals to generate HO^•^. We also present a systematic analysis of six different metal ferrites (MFe_2_O_4_, M = Mg, Fe, Co, Ni, Cu, and Zn) with no surface ligands to analyze the effects that changes in composition may have on the activity of this type of material toward photoinduced degradation of methyl orange via the photo-Fenton process. We describe the removal efficiency in terms of adsorption under dark conditions and photoassisted degradation. We compare the degradation percentages with previous reports and against commercially available anatase TiO_2_ nanoparticles as a reference material to benchmark the performance of our materials. We confirm that the ability of a metal ferrite to induce degradation of methyl orange under irradiation correlates with the efficiency with which it forms HO^•^ radicals, which we detect using a fluorescence assay. On the other hand, the observed adsorption of methyl orange to the surface of the nanomaterial correlates with the magnitude of the surface charge of the metal ferrite nanocrystals, which we estimate using the difference between the pH of the reaction mixture and the pH at the point of zero charge (pH_pzc_) of the nanomaterial. We therefore attribute the adsorption behavior to electrostatic interactions between the nanocrystal surface and the charged methyl orange molecule. These data indicate that examination of surface chemistry is crucial to understanding the performance of spinel ferrite nanomaterials in photo-Fenton chemistry.

## Experimental Methods

### Synthetic Methods

#### Synthesis of Colloidal NiFe_2_O_4_ Nanocrystals

Colloidal NiFe_2_O_4_ nanocrystals were synthesized using our previously reported method.^36^ Briefly, NiFe_2_(μ_3_-O)(μ_2_-O_2_CCF_3_)_6_(H_2_O)_6_ (0.025 mmol, ∼23 mg), oleic acid (OA, 2.7 mmol, 0.848 g), oleylamine (OAm, 2.7 mmol, 0.737 g), and benzyl ether (10 mL, 10.43 g) were added to a 25 mL Teflon insert. The mixture was magnetically stirred for 10–15 min at 500 rpm under ambient conditions to form a clear dark red/brown suspension. Subsequently, the Teflon insert was capped, loaded into a stainless-steel autoclave, sealed, and heated at 230 °C for 24 h. After 24 h, the autoclave was allowed to cool over a period of 4–6 h (or overnight) under a well-ventilated fume hood. The suspension was then purified with three cycles of precipitation with ethanol followed by centrifugation.

#### Ligand Exchange

A 0.01 M solution of ligand (nitrodopamine, mercaptosuccinic acid, citric acid, or (aminomethyl)phosphonic acid) in 5 mL of dimethylformamide (DMF) was prepared via sonication for 5 min. A single batch of as-synthesized NiFe_2_O_4_ NCs was dissolved in 5 mL of hexane and added to the DMF ligand solution. The mixture was then sonicated for 3 h at a temperature of 40 °C. After that period, phase transfer of the nanocrystals from hexane to DMF was readily visible. The upper layer (clear hexane layer) was then removed, and the remaining colloidal dispersion was washed with cold methanol 3 times to remove excess ligands.

#### Synthesis of Ligandless MFe_2_O_4_ (M: Fe, Co, Ni, and Zn) Nanomaterials

MFe_2_O_4_ nanoparticles (M = Co, Ni, and Zn) were synthesized using stoichiometric (2:1) mixtures of Fe(acac)_3_ (2.8 mmol) and M(acac)_2_ (1.4 mmol) dissolved in 40 mL of benzyl ether. For Fe_3_O_4_, 2.6 mmol of Fe(acac)_3_ dissolved in 40 mL of benzyl ether was used as the sole precursor. After stirring for 30 min at room temperature, the reaction mixtures were heated to 230 °C for 24 h in a 100 mL autoclave reactor. The autoclave was allowed to cool over a period of 8–12 h under a well-ventilated fume hood. The resulting nanocrystals were then purified with three cycles of precipitation with ethanol followed by centrifugation.

#### Synthesis of Ligandless MgFe_2_O_4_ Nanomaterials

A 2:1 mixture of Mg(acetate)_2_·4H_2_O (2.5 mmol) and Fe(NO_3_)_3_·9H_2_O (5 mmol) was combined with sodium acetate (10 mmol) and 35 mL of ethylene glycol. After sonicating for 1 h at room temperature, the mixture was heated to 200 °C for 11 h in a 100 mL autoclave reactor. The autoclave was allowed to cool over a period of 8–12 h under a well-ventilated fume hood. The resulting nanocrystals were then purified with three cycles of precipitation with ethanol followed by centrifugation.

#### Synthesis of Ligandless CuFe_2_O_4_ Nanomaterials

A 2:1 mixture of Cu(NO_3_)_2_·3H_2_O (1 mmol) and Fe(NO_3_)_3_·9H_2_O (2 mmol) was combined with sodium acetate (15 mmol) in 30 mL of ethylene glycol. The mixture was stirred overnight to ensure thorough mixing before heating to 180 °C for 12 h in a 100 mL autoclave reactor. The autoclave was allowed to cool over a period of 8–12 h under a well-ventilated fume hood. The resulting nanocrystals were then purified with three cycles of precipitation with ethanol followed by centrifugation.

### Nanomaterials Characterization

#### Transmission Electron Microscopy (TEM)

TEM micrographs were obtained using a FEI Tecnai F20 transmission electron microscope with a beam energy of 200 kV. The nanocrystal samples were drop-cast from hexane onto copper grids coated with lacy carbon.The diameter of the particles was measured using ImageJ software (version 1.52a).^37^

#### Scanning Electron Microscopy (SEM)

SEM micrographs were obtained using a Zeiss Auriga scanning electron microscope with a beam energy of 25 kV. The metal ferrite samples were drop-cast onto silicon wafers from hexane dispersions.

#### Powder X-ray Diffraction

We performed powder X-ray diffraction measurements on dried nanocrystal powders using a Rigaku XtaLAB Dualflex Synergy-S diffraction system with Mo Kα radiation (λ = 0.71073 Å). We converted the 2θ values obtained using the Mo source to 2θ values corresponding to the wavelength of a Cu Kα source (λ = 1.54148 Å) to compare our measured spectra to standard data deposited in the JCPDS database that was collected with Cu Kα radiation.^36^

#### X-ray Photoelectron Spectroscopy (XPS)

XPS measurements were performed on three separate samples of each nanocrystal batch to ensure data reproducibility. Sample preparation was performed under an ambient atmosphere. The nanocrystal powders were dissolved in hexane to obtain a concentrated solution. The solution was drop-cast onto cleaned Si wafers, which were electrically grounded to the sample bar by carbon tape. The XPS measurements were recorded with a Kratos Axis Ultra DLD system equipped with a monochromatic Al Kα (*h*ν = 1486.6 eV) X-ray source. During the measurements, pressure in the main chamber was kept below 1 × 10^–7^ mbar. Charge compensation was performed via a neutralizer running at a current of 7 × 10^–6^ A, a charge balance of 5 eV, and a filament bias of 1.3 V. The X-ray gun was set to 10 mA emission. Binding energies were referenced to the C 1s peak arising from adventitious carbon with binding energy of 284.8 eV. The C 1s, O 1s, Fe 2p, and M 2p core levels were recorded with a pass energy of 80 eV. We collected three scans for iron, M, and oxygen and two scans for carbon. XPS analysis was performed with CasaXPS (version 2.3.22PR1.0.) The *U* Touggard function was used for background subtraction, and the peaks were fit with one or more Gaussian components. The XPS signals were fitted with the CasaXPS Component Fitting tool.

#### Energy Dispersive X-ray Spectroscopy (EDS)

EDS elemental information was obtained using a Zeiss Auriga scanning electron microscope coupled to an EDS analyzer. Measurements were performed using a 25 kV electron beam energy. Semiquantitative data analyses were performed using the EDAX Apex software.

#### Atomic Absorption Measurements

Solutions of NiFe_2_O_4_ were digested in a 0.2% nitric acid solution in Nanopure water. Solutions with known iron concentrations ranging from 0 to 6 ppm were prepared by diluting a standard iron solution (100 ppm from High-Purity Standards) with a 0.2% nitric acid solution in Nanopure water. Atomic absorption (AA) measurements were performed in a Shimadzu atomic absorption spectrophotometer (AA-7000 series) using a hollow cathode Fe lamp. The Supporting Information contains details of the calibration procedures and data obtained from these measurements.

#### Fourier Transform Infrared (FTIR) Spectroscopy

A PerkinElmer Spectrum 3 FTIR spectrophotometer equipped with a diamond ATR crystal was used to record all infrared spectra in attenuated total reflection mode [FT-IR (ATR)], and these data are reported in wavenumbers (cm^–1^).

#### ^1^H NMR Spectroscopy

^1^H NMR spectra were collected on a 400 MHz Brüker spectrometer. Samples containing nanocrystals functionalized with oleic acid and oleylamine were dispersed in fully deuterated toluene, while all other samples were dispersed in D_2_O.

### Photoinduced Degradation of Methyl Orange

We utilized a Xe Arc lamp (optical power = 0.8 Wcm^–2^) for all photoinduced degradation experiments, and all the experiments were performed at room temperature (RT: 21 °C). Scheme S1 contains a schematic of our photoreaction setup. For the photodegradation studies, 10 mg of ferrite nanocrystals was sonicated in 20 mL of methyl orange solution (10 mg/L in Nanopure water) and placed inside a 25 mL scintillation vial. The vial was agitated on a SK-O180-S ONiLAB digital orbital shaker for at least 24 h in the dark, during which time samples of the reaction mixture were periodically extracted and characterized by UV–vis spectroscopy using the sampling technique reported below. After reaching equilibrium in the dark, 13 μL of 30% H_2_O_2_ solution was added to the reaction mixture and allowed to mix on the orbital shaker for 15 min. Subsequently, 1.5 mL of the aqueous phase was pulled out of the vial through a disposable syringe filter (pore size: 0.2 μm) to lock the nanocrystals. The locked nanocrystals were pushed back into the aqueous solution by pushing 0.5 mL of the aqueous phase back through the syringe. The remaining 1 mL of the sample solution was analyzed by UV–vis spectroscopy. This sample corresponds to *t* = 0. The reaction mixture was then placed in front of a water filter arranged 30 cm away from both the sample and the Xe Arc lamp (spot size: 6.8 cm^2^). The cutoff wavelength for the water filter is 350 nm (see Figure S1). Samples were periodically extracted from the reaction mixture using the process described above and analyzed by UV–vis spectroscopy to track the progress of the degradation reaction. Control experiments to test the role of H_2_O_2_ in the photodegradation reaction were completed using the same procedure, but without addition of H_2_O_2_.

### Use of Terephthalic Acid as a Fluorescent Probe to Detect HO^•^ Radical Formation

We mixed 10 mg of ferrite nanocrystals with 20 mL of a 7 mM solution of terephthalic acid (TPA) in Nanopure water. Experiments were conducted under four different conditions: (1) with H_2_O_2_ and illumination, (2) without H_2_O_2_, but with illumination, (3) with H_2_O_2_ in the dark, and (4) without H_2_O_2_ in the dark. For experiments containing H_2_O_2_, 13 μL of 30% H_2_O_2_ (6.4 mM) was added to the reaction mixture. For experiments performed with illumination, the samples were exposed to the output of the filtered Xe arc lamp (0.8 W cm^–2^) for 5 h prior to characterization by fluorescence. For experiments performed with H_2_O_2_ in the dark (3), the nanocrystals were allowed to sit with H_2_O_2_ and TPA in the dark for 5 h prior to fluorescence measurements to mimic the duration the nanocrystals were exposed to H_2_O_2_ during photodegradation experiments. For experiments performed without H_2_O_2_ in the dark, the samples were allowed to sit with TPA in the dark for 16 h. To characterize the mixtures via fluorescence, the aqueous mixture was pulled out of the reaction vial, passed through a disposable syringe filter, and placed in a 1 cm path length cuvette for further analysis. Photoluminescence characterization was completed with a modular Acton fluorometer setup using a photomultiplier tube detector. Fluorescence measurements were collected over the range of 380–600 nm following 360 nm excitation. Solutions containing a known fluorescent product of the reaction of HO^•^ with TPA, hydroxyterephthalic acid (hTPA) in Nanopure water, were used as references for spectral comparison.

## Results and Discussion

### Surface Functionalization Impedes Photocatalytic Activity of NiFe_2_O_4_ Nanocrystals

In general, colloidal spinel ferrites, and many other metal oxide nanocrystals, are synthesized in the presence of surfactants with long aliphatic chains, such as oleic acid^38,39^ and oleylamine.^36,40−42^ The nonpolar and sterically bulky nature of these surface ligands provides nanoscale materials with colloidal stability in nonpolar solvents; however, in quantum-dot colloidal nanocrystals, these ligands have been observed to prevent the adsorption of small molecule substrates.^43^ Therefore, aiming to increase the colloidal stability of metal ferrite nanocrystals in aqueous media and improve the permeability of the ligand shell to the pollutants of interest, we exchanged the native surface ligands for ligands that have shorter and more polar side chains.

Inspired by the exciting results from Gupta et al.,^23^ where nickel ferrite was found to outperform cobalt, copper, and zinc ferrites in the photodegradation of a series of dye molecules, we examined the photocatalytic activity of a series of four different ligand-functionalized NiFe_2_O_4_ nanocrystals, namely NiFe_2_O_4_-NDA (NDA: nitrodopamine), NiFe_2_O_4_-MSA (MSA: mercaptosuccinic acid), NiFe_2_O_4_-CA (CA: citric acid), and NiFe_2_O_4_-AMPA (AMPA: (aminomethyl)phosphonic acid). The choice of these ligands was inspired by Deblock et al.,^44^ who established a protocol for ligand exchanges applicable to metal oxide NCs, in particular HfO_2_ nanocrystals, and assessed how well these ligands interacted with the NC surfaces in polar solvents.

**Figure 2:**
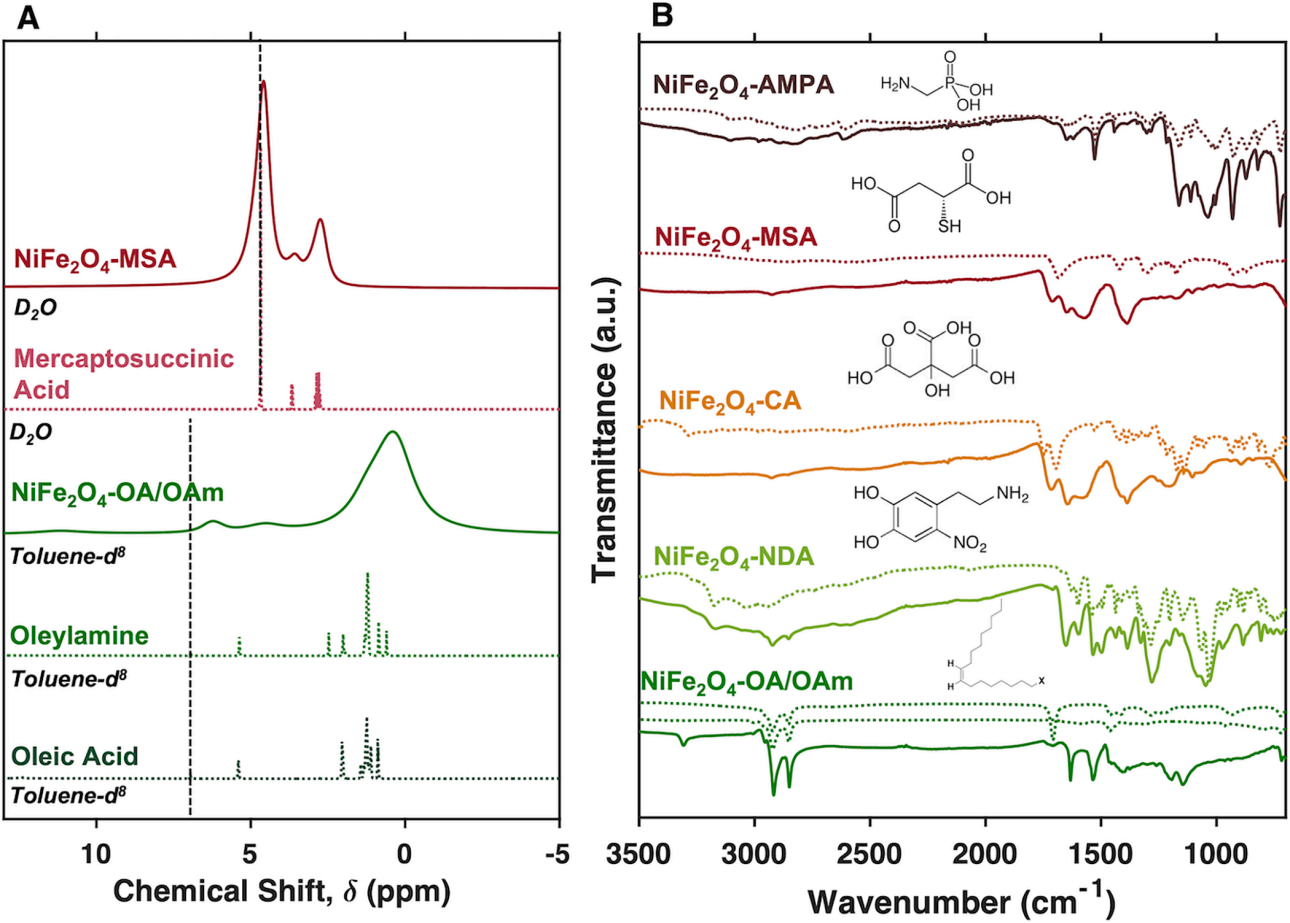
(A) Representative ^1^H NMR spectra of the successful ligand exchange of OA/OAm ligands in NiFe_2_O_4_ for mercaptosuccinic acid. Spectra of pre- and post-exchanged NiFe_2_O_4_ nanocrystals are plotted as solid lines (green and maroon, respectively), and specra of pure ligands are plotted as dotted lines (MSA - pink, OAm - green, OA - dark green). The dashed vertical lines indicate the position of the solvent peak. Analogous ^1^H NMR spectra characterizing the ligand exchange of OA/OAm for citric acid, nitrodopamine, and (aminomethyl)phosphoric acid are included in Figure S3. (B) FTIR spectra of surface-functionalized NiFe_2_O_4_. The plotted dotted lines illustrate the FTIR of the corresponding pure ligands.

We synthesized NiFe_2_O_4_ nanocrystals (NCs) from a single-source precursor in the presence of oleic acid (OA) and oleylamine (OAm) in benzyl ether (BE) at 230 °C in a solvothermal reactor following our previous report.^36^ The resulting NiFe_2_O_4_ NCs form stable colloidal dispersions in nonpolar solvents, particularly hexanes and toluene. The quasicubic nanocrystals present a diameter of 10.3 ± 1 nm (*x̅* ± σ) (Figure S2) and possess face-centered cubic symmetry that belongs to the space group *Fd*3*m* according to powder XRD (Figure S3), which is consistent with the spinel crystal structure. Representative ^1^H NMR spectra of NiFe_2_O_4_ nanocrystals collected after ligand exchange with mercaptosuccinic acid (MSA) are shown in Figure 2A. These spectra exhibit peaks associated with the added MSA with significant broadening due to the combined effects of the paramagnetism of our samples and the steric restriction imposed upon surface-bound ligands that elongates T_2_ (see Figure S4). We also observe the complete disappearance of the NMR signals of native ligands (oleic acid and oleylamine, Figure 2A). We used ATR-FTIR to further confirm the success of our ligand exchange reactions (Figure 2B). We observed that the OA/OAm ligand substitutions by NDA, CA, MSA, and AMPA are stable throughout a series of washing steps as confirmed by ATR-FTIR (Figure 2B), where the original OA/OAm features disappear and the newly added ligands’ absorbance bands shift and/or broaden (compared to free ligands) after the surface ligand exchange. Notably, however, we observed that the NiFe_2_O_4_-AMPA NCs did not remain colloidally stable in water after more than 10 min.

**Figure 3:**
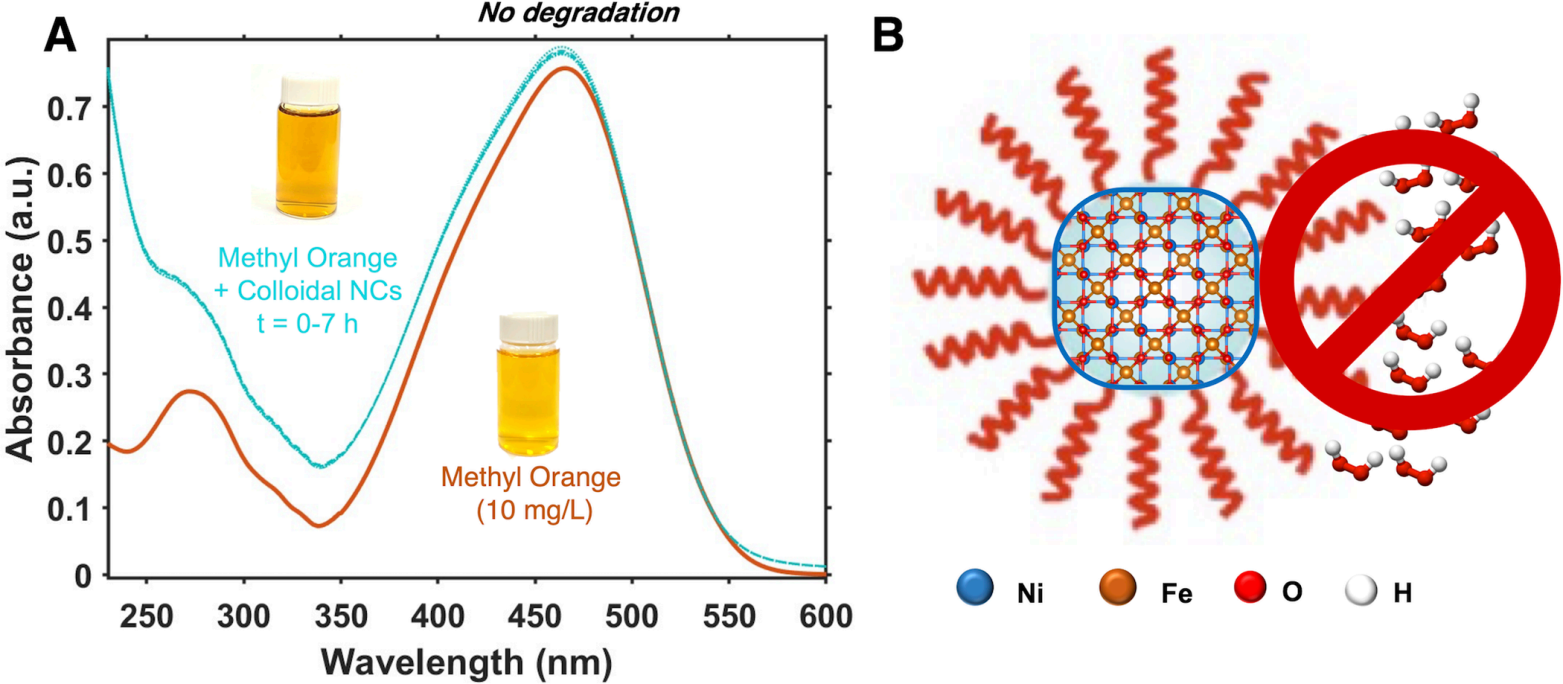
(A) Representative UV–vis absorption spectra demonstrating minimal concentration changes in the methyl orange solution over time under the photocatalytic reaction conditions in the presence of NiFe_2_O_4_-MSA nanocrystals corresponding to 250 mol % NiFe_2_O_4_ relative to methyl orange and 6.4 mM H_2_O_2_. (B) Schematic representation of ligands blocking the access of hydrogen peroxide molecules to the nanocrystal surface.

Aiming to understand the reasons behind the superstoichiometric nanocrystal loads that are ubiquitous in previous reports investigating the use of spinel ferrites to induce photodegradation (Table 1), we first tested our nanocrystals under substoichiometric loadings to determine if such nanocrystal concentrations would provide the necessary reactivity to drive the degradation reactions. We decided to first conduct our reactions using a 25 mol % loading of NiFe_2_O_4_ (see Figure S6 and Table S2 for details of how these loadings were quantified). However, this concentration of nanocrystals did not induce any degradation of methyl orange. Therefore, we increased the catalyst dose by an order of magnitude (up to 250 mol % of NiFe_2_O_4_) to assess if the lack of activity was due to the low content of surface reactive sites. Unfortunately, as observed in Figure 3A and Figure S7, no evidence of photocatalytic degradation was observed despite significant absorption of visible light by the colloidal NiFe_2_O_4_ nanocrystals (see Figures S8 and S9). We hypothesized that the surface ligands, although rendering colloidal stability in water, decrease the ability of the hydrogen peroxide molecules to access the nanocrystal surface (Figure 3B) and thereby inhibit the electron–hole pair redox chemistry at the surface that produces reactive oxygen species.

**Figure 4:**
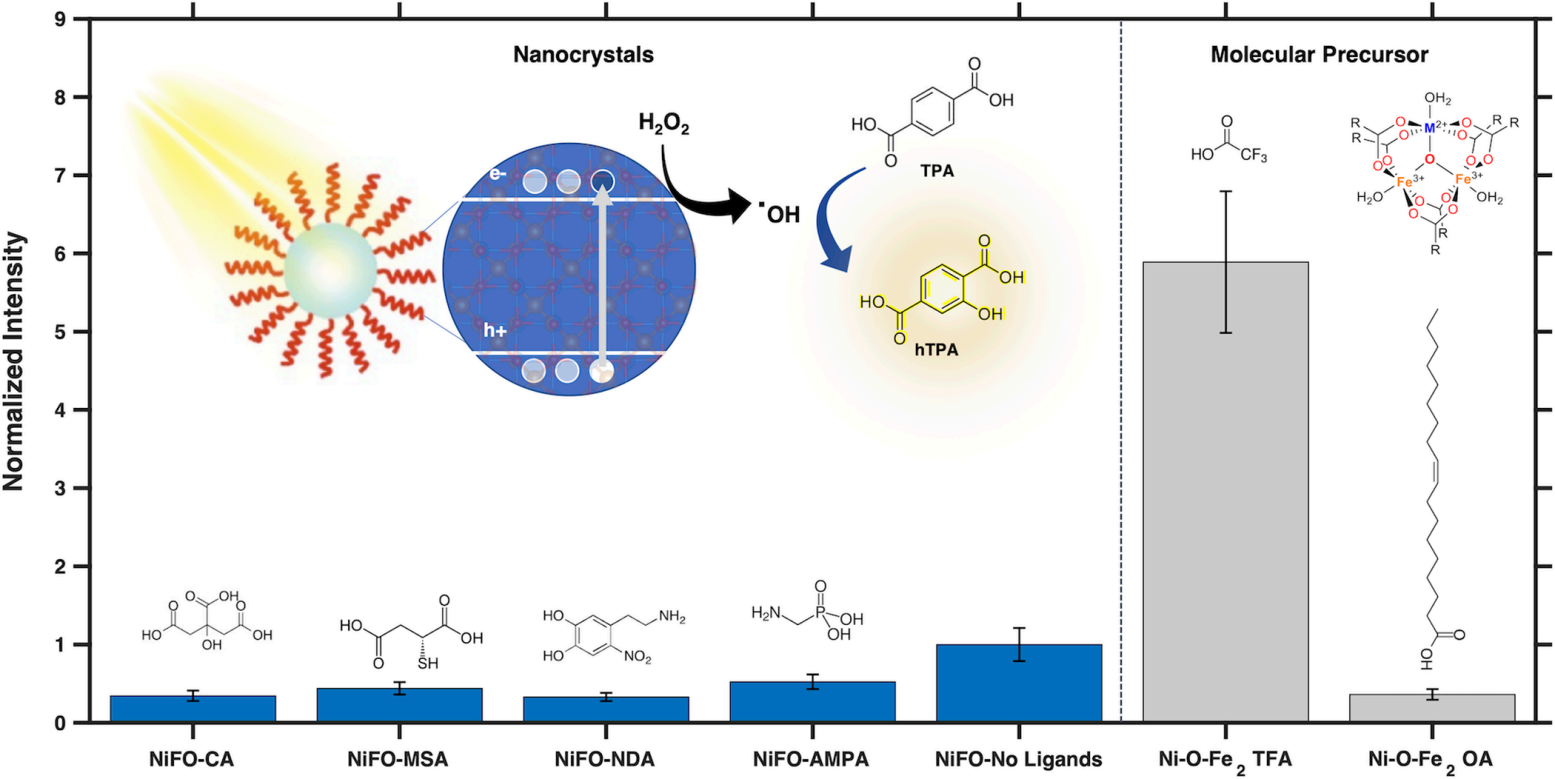
Bar graph showcasing the formation of fluorescent products upon illumination of functionalized (colloidal) and bare NiFe_2_O_4_ nanocrystals (blue) as well as Ni–O–Fe_2_ single-source precursor molecules functionalized with trifluoroacetic acid (TFA) or oleic acid (OA) in the presence of 7 mM terephthalic acid (TPA) and 6.4 mM H_2_O_2_ for 5 h. The error bars originate from the standard deviation of three trials, and the intensities are normalized to that observed for the ligandless NiFe_2_O_4_ nanocrystals (see the Supporting Information). The left inset showcases the overall mechanism of radical generation and detection through hTPA formation as the primary fluorescent product.

We explored this hypothesis by studying the formation of hydroxyl radicals directly using a fluorescent assay for HO^•^, namely terephthalic acid (TPA; see Figure S8 for representative fluorescence spectra and additional discussion of this fluorescence assay method).^23,45−52^Figure 4 displays the integrated fluorescence intensity detected in colloidal solutions of NiFe_2_O_4_ nanocrystals corresponding to iron concentrations of 0.306 μM after 5 h of light exposure in the presence of 6.4 mM H_2_O_2_ and 7 mM TPA. This nanocrystal concentration is the same concentration used in the photocatalysis experiment shown in Figure 3A containing 250 mol % NiFe_2_O_4_ relative to methyl orange. Out of the four types of surface-functionalized NiFe_2_O_4_ NCs evaluated in this assay, (NiFO-NDA, NiFO-MSA, NiFO-CA, and NiFO-AMPA), AMPA generates the highest fluorescence intensity; however, the amount of HO^•^ produced under these conditions is apparently not sufficient to result in a detectable change in the concentration of methyl orange. As previously mentioned, this set of NiFO-AMPA NCs does not remain colloidally stable for prolonged periods; it tends to “crash out” of solution. Hence, we suspect that this unstable ligand shell may allow accessible reactive sites and surface-trapped charge carriers to interact more closely with hydrogen peroxide molecules, which may in turn explain the higher observed fluorescence intensity.

With this idea in mind, we performed a series of control experiments including both NiFe_2_O_4_ nanocrystals synthesized in the absence of oleic acid and oleylamine and the single-source precursor, NiFe_2_(μ_3_-O)(μ_2_-OOCR)_6_(OH_2_)_3_, to test the hypothesis that surface-bound ligands inhibit the photoinduced generation of HO^•^. We used two versions of the single-source precursor molecule, one with trifluoroacetate ligands (R = CF_3_, denoted as Ni–O–Fe_2_ TFA) and one with oleate ligands (R = CH_3_(CH_2_)_7_CH=CH(CH_2_)_7_, denoted as Ni–O–Fe_2_ OA), to model the impact of surface steric effects on the generation of hydroxyl radicals. We observed that bare NiFe_2_O_4_ nanocrystals(size: 8 ± 1.0 nm; 774 Fe-surface sites per nanocrystal) can produce about twice the fluorescence intensity as NiFO-AMPA due to reaction of HO^•^ with TPA. We corroborate this finding by evaluating the impact of tuning the ligands in the single-source precursor from short chain polar to long chain nonpolar carboxylates on the formation of HO^•^ radicals. We observe that increasing the steric bulk of the ligand on the single-source precursor by switching from trifluoroacetate to oleate impedes the photoinduced formation of HO^•^ by a factor of ∼40. We also observe that Ni–O–Fe_2_ TFA produces significantly more fluorescence signal than bare NiFe_2_O_4_ nanocrystals. Since the total concentrations of Ni and Fe in the NiFe_2_O_4_ and Ni–O–Fe_2_ experiments were kept the same, we attribute the observed increase in formation of HO^•^ in the presence of Ni–O–Fe_2_ compared to NiFe_2_O_4_ to the fact that all of the metal cations in the molecule are accessible to H_2_O_2_ whereas a significant fraction of the Ni and Fe atoms in NiFe_2_O_4_ nanocrystals are sequestered in the nanocrystal core. These results inspired us to analyze the potential reactivity of the trifluoroacetate molecular complex toward methyl orange degradation; however, no degradation was observed within 5 h of irradiation (Figure S6D), leading us to conclude that under these conditions, the amount of HO^•^ generated is not enough to promote detectable degradation of methyl orange molecules in solution.

### Nonfunctionalized Pristine Surfaces Improve the Ability of Metal Ferrite Nanocrystals to Remove Methyl Orange from Solution

Motivated by the results from the previous section, we decided to synthesize a series of spinel ferrite nanomaterials (MFe_2_O_4_), with M = Mg, Fe, Co, Ni, Cu, and Zn, without surface ligands to investigate the impact of spinel composition on photocatalytic activity. Changes in composition are known to impact the band-edge potentials and band gaps of metal oxide materials^53^ and may also affect the lifetime of photogenerated charge carriers and rates of surface redox processes that generate the reactive oxygen species (ROS) that degrade the pollutant in solution. However, the relationship between the bulk composition of a metal ferrite nanocrystal and its surface chemistry and subsequent impacts on its ability to mediate photoinduced degradation of organic molecules has not been explored in detail.

**Figure 5:**
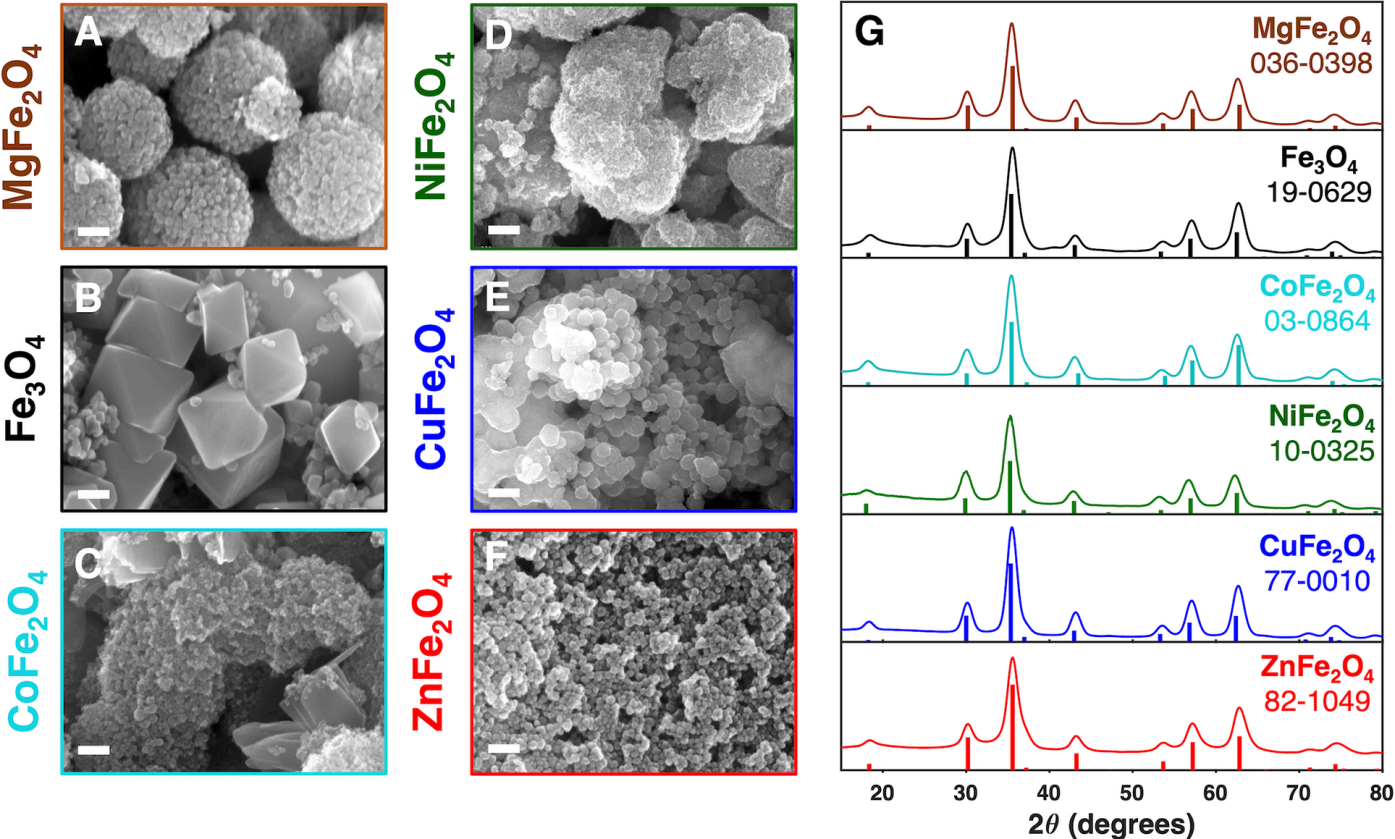
(A–F) Representative SEM micrographs of nanostructured metal ferrites (scale bars = 100 nm). (G) Powder X-ray diffractograms of each metal ferrite labeled with its JCPDS reference number.

Figure 5G contains the powder X-ray diffraction (XRD) spectra obtained for this series of spinel ferrite nanomaterials, which confirm their bulk phase purity. The surface morphology, stoichiometry, and chemistry of each ferrite were analyzed via scanning electron microscopy (SEM), X-ray photoelectron spectroscopy (XPS), and FTIR techniques. Figure 5A-F showcases the overall hierarchical morphology of the obtained nanomaterials. Except for Fe_3_O_4_, which presents an octahedron-like morphology with well-defined facets, these metal ferrite nanomaterials comprise aggregates formed by quasi-spherical crystallites and possess some degree of surface roughness, which is a general characteristic of these materials in the bulk.^54−56^Figure S11 displays the energy-dispersive X-ray spectra (EDS) of all ferrites, confirming the presence of all expected elements.

Photodegradation experiments were performed using superstoichiometric amounts of ferrite nanocrystals relative to methyl orange (see Table 2 for mol % values and Table S3 for details on how these values were calculated). We chose to use such large amounts of ferrite for two reasons: (*i*) this amount represents the average amount of ferrite used in previous reports (0.5 g/L) and so facilitates comparison of our results with those from previous reports; (*ii*) we do not observe significant photodegradation of methyl orange in the presence of smaller amounts of metal ferrite (Figure 3). In the absence of metal ferrite nanocrystals, ∼2% dye degradation was observed after 5 h in the presence of H_2_O_2_ in the dark. In the presence of light and H_2_O_2_, but in the absence of metal ferrite nanocrystals, ∼5% degradation was observed over 5 h. Data from these control experiments are plotted in Figure S12. We suspect this nominal degradation arises from photoreduction of H_2_O_2_ by methyl orange, which is enabled by the overlap between the methyl orange absorption spectrum and the lamp spectrum in the 400–600 nm region (Figure S8). In the absence of both H_2_O_2_ and metal ferrite nanocrystals no degradation was found at all under illumination (Figure S12).

**Table 2:** Percent of Methyl Orange Removed by Adsorption in the Dark and Photoinduced Degradation in the Presence of the MFe_2_O_4_ Nanomaterials^a^

material	adsorption (%) ± STD	photoinduced degradation (%) ± STD	total removal (%)	mol % MFe_2_O_4_
MgFe_2_O_4_		52 ± 1	52 ± 1	8200
Fe_3_O_4_		96 ± 1	96 ± 1	7100
CoFe_2_O_4_	29 ± 1	20 ± 1	49 ± 2	7000
NiFe_2_O_4_	87 ± 3		87 ± 3	7000
CuFe_2_O_4_	88 ± 2	8 ± 1	96 ± 3	6900
ZnFe_2_O_4_	33 ± 3		33 ± 3	6800
w-TiO_2_		18^b^	18^b^	20500
		98^c^	98^c^	

a The standard deviations originate from data obtained in three trials of the same reaction.

b With water filter.

c Without water filter.

**Figure 6:**
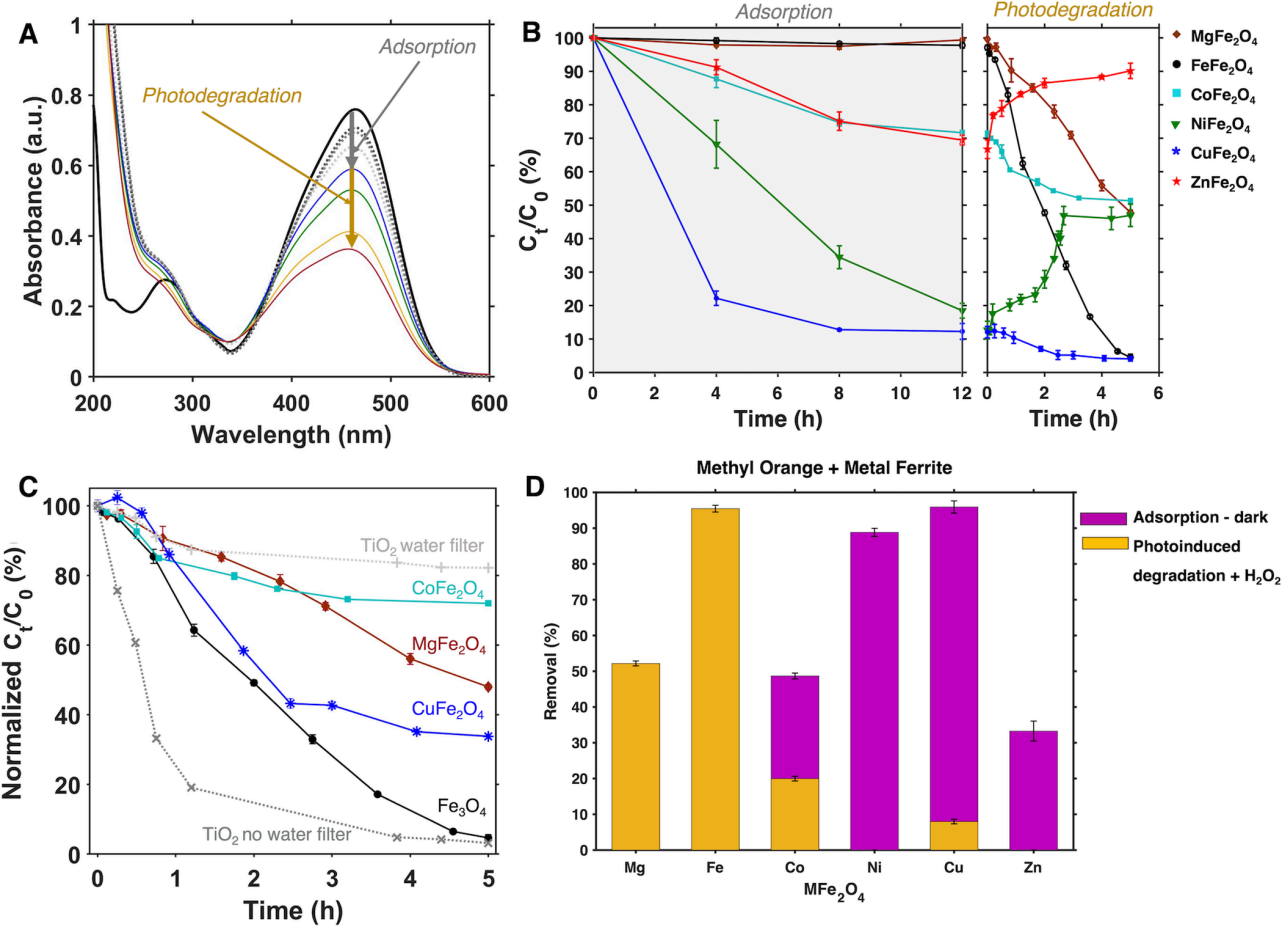
(A) Representative absorption spectra collected of samples containing ligandless CoFe_2_O_4_ nanocrystals, methyl orange, and H_2_O_2_ at various time points after the samples were prepared. The absorption spectrum of a 10 ppm solution of pure methyl orange is plotted in black. The dotted lines indicate spectra collected during the initial adsorption equilibration period in the dark, and the solid lines indicate spectra collected during the illumination period. (B) Kinetics of adsorption over a period of 12 h in the dark in the absence of H_2_O_2_ (left) and subsequentdegradation of methyl orange under illumination in the presence of H_2_O_2_ (right). Each samplespent at least 24 h in the dark prior to addition of H_2_O_2_ and illumination. (C) Kinetics of photodegradation normalized to 100% at *t* = 0 h under illumination for comparison purposes. (D) Total percentage of methyl orange removal in the dark (magenta bars) and photoinduced degradation (yellow bars).

Overall, we observe two different processes by which the spinel ferrite nanomaterials remove methyl orange from solution: (*i*) adsorption in the dark and (*ii*) photocatalytic degradation. Figure 6A displays a representative experiment of the adsorption and photodegradation of methyl orange tracked via UV–vis spectroscopy (gray and rainbow colors, respectively; additional spectra are plotted in Figure S13). In the absorption spectrum of methyl orange (Figure 3A) there are two main absorption peaks. The high-intensity peak at 464 nm is due to a π → π* transition from the azo N=N functionality, the molecule’s chromophore.^57^ The lower-intensity peak in the ultraviolet region (275 nm) appears due to the aromatic rings. When the photodegradation occurs in the presence of metal ferrite nanocrystals and H_2_O_2_, the intensity of the peaks at both 464 and 275 nm decreases with time without forming any new band in the UV or visible region. This observation is consistent with degradation of both the azo functional group^58,59^ and the aromatic rings, but not sufficient to conclude that complete mineralization of all organic components has occurred upon decolorization. Previous studies of methyl orange degradation employing total organic carbon (TOC) analysis demonstrate that decolorization is associated with loss of organic carbon, indicating the formation of inorganic products, which would be consistent with mineralization.^60,61^ One recent study of methyl orange photodegradation by surface-modified Fe_3_O_4_ nanoparticles in the presence of H_2_O_2_ demonstrated that the colorless degradation products are significantly less toxic to fish than methyl orange.^62^ Given the similarity of this photodegradation approach with the spinel ferrite photodegradation systems that are the subject of this Perspective, we strongly suspect that the colorless degradation products produced here are also primarily nontoxic mineralization products.

Figure 6B showcases three different scenarios observed for different metal ferrite nanocrystals: (I) photoinduced degradation but no observable adsorption in the dark (MgFe_2_O_4_, Fe_3_O_4_), (II) adsorption and photoinduced release of adsorbed species (NiFe_2_O_4_, ZnFe_2_O_4_), and (III) adsorption and photoinduced degradation activity (CoFe_2_O_4_, CuFe_2_O_4_). We note that those ferrites that induce significant removal of methyl orange in the dark do so over at least 12 h of the dark period. These results indicate that the 30 min–1 h “adsorption–desorption” equilibration time used in most of the studies highlighted in Table 1 may not be sufficient to achieve equilibrium. Table 2 tabulates the percentages of the initial concentration of methyl orange that are removed by adsorption and photoinduced degradation for each metal ferrite; Figure 6D depicts these data graphically. We found that MgFe_2_O_4_ and Fe_3_O_4_ belong to scenario I. These materials do not exhibit any significant adsorption of methyl orange over a period of >24 h in the dark but induce degradation of methyl orange after 5 h of illumination by 52 and 96%, respectively. On the other hand, NiFe_2_O_4_ and ZnFe_2_O_4_ belong to scenario II, adsorbing almost 89 and 33% of the total methyl orange concentration available from the initial concentration and photoreleasing 39 and 23% adsorbed methyl orange back to solution, respectively. CoFe_2_O_4_ and CuFe_2_O_4_ belong to scenario III: they display adsorption of methyl orange overnight of 29 and 88%, and they photodegrade 20 and 8% of the initial concentration of methyl orange, removing about 49 and 96% of the total methyl orange concentration, respectively.

In Figure 6C, the kinetics of photoinduced degradation of methyl orange in the presence of metal ferrites from scenarios I and III are normalized to 100% at *t* = 0 for comparison purposes. We find that Fe_3_O_4_ is the most efficient ferrite material for inducing methyl orange removal under illumination, removing up to 95% of the methyl orange present at a nearly constant rate. MgFe_2_O_4_ also removes methyl orange at a roughly constant, but slower, rate whereas both CoFe_2_O_4_ and CuFe_2_O_4_ have rates that slow down over the 5 h reaction period. As a standard reference to discuss the efficacy of these materials to degrade pollutants upon visible light excitation, we include data collected for commercial anatase TiO_2_ nanocrystals. We note that compared to TiO_2_ exposed to the filtered output of the Xe arc lamp (λ > 350 nm), our materials present larger removal efficiencies (Table 2 and Figure 6C); however, when TiO_2_ is exposed to the full spectrum of the xenon arc lamp, which includes higher frequency UV light, the removal efficiency is equivalent to that of our best performer, Fe_3_O_4_, with an increased degradation rate (Figure 6C). These results demonstrate that the metal ferrite nanomaterials used here actively induce degradation of methyl orange under visible light irradiation.

To investigate the origins of the different photodegradation activities, we characterized the optical properties of the metal ferrite nanomaterials in powder form as well as dispersed in water at the same concentrations used for the photodegradation measurements. We estimated the position of the absorption onset of the metal ferrite nanomaterials from diffuse reflectance measurements of films drop-cast on glass slides (Figure S14). All the ferrites have absorption onsets less than or equal to 1.8 eV (∼700 nm), indicating that all of these materials are capable of absorbing the majority of the visible spectrum. However, we found no correlation between the absorption onset and the photodegradation activity (see Figure S14). We also characterized the total transmission of the filtered output of the Xe arc lamp through the various ferrite dispersions. As shown in Figure S15, all of these dispersions exhibit visible aggregates that would be expected to induce large scattering. Indeed, we suspect that the bulk of the transmission loss observed through the ferrite dispersions arises due to scattering. We also observe a minimal correlation between the transmission through these dispersions and the ability of these nanocrystals to induce photodegradation of methyl orange. These data suggest that differences in the optical properties are not sufficient to account for the differences in the photoreactivity toward degradation.

**Figure 7:**
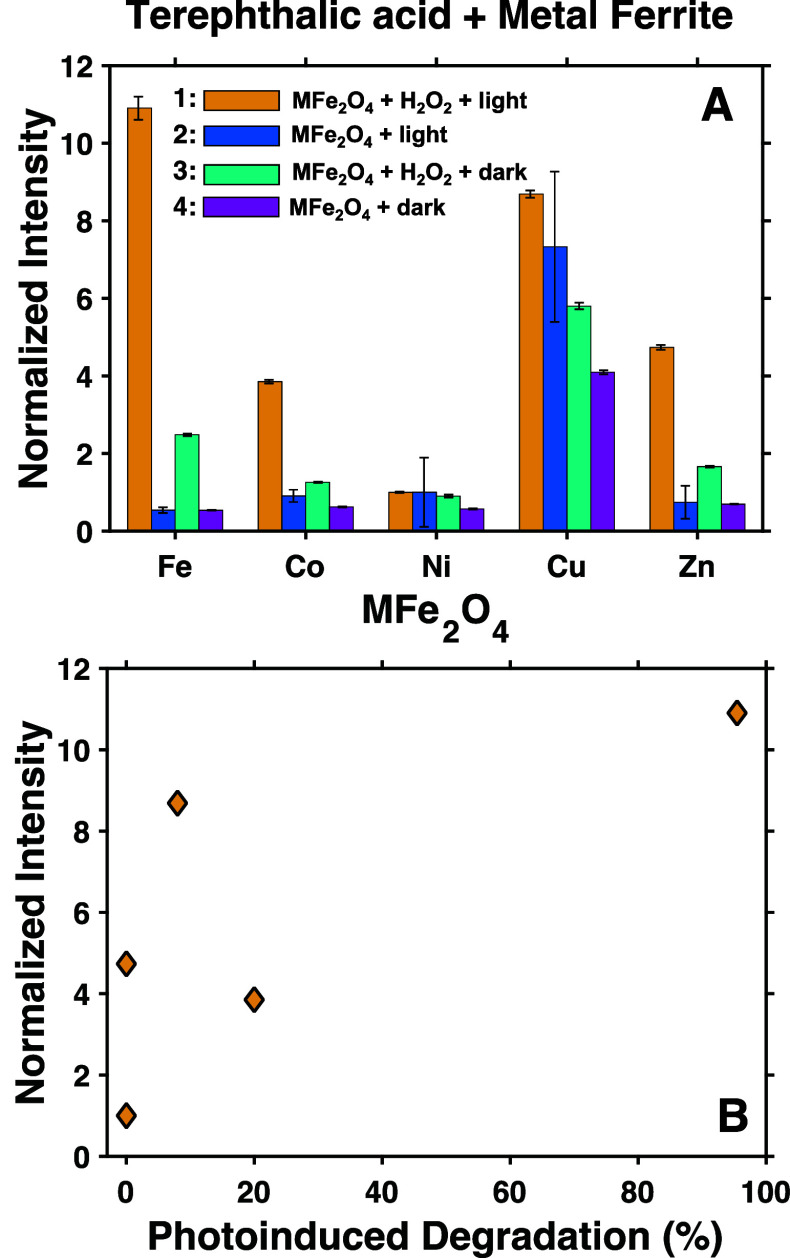
(A) Appearance of fluorescent products upon exposure of ferrite nanocrystals to TPA under four different conditions: (1) MFe_2_O_4_ + H_2_O_2_ + TPA + light (yellow bars); (2) MFe_2_O_4_ + TPA + light (dark blue bars); (3) MFe_2_O_4_ + H_2_O_2_ + TPA in the dark (light blue bars); (4) MFe_2_O_4_ + TPA in the dark (pink bars). Intensities are normalized to that observed for the NiFe_2_O_4_ nanocrystals under condition 1 (see the Supporting Information). (B) Plot of normalized integrated fluorescence intensity obtained from illuminated mixtures containing MFe_2_O_4_, H_2_O_2_, and TPA (condition 1) vs the percent of methyl orange removed under illumination.

To further investigate why some of the metal ferrites studied here present higher activity for photoinduced degradation of methyl orange than other ferrites, we analyzed their capability to generate hydroxyl radicals via the TPA fluorescent assay. Figure 7A displays the relative fluorescence intensity detected under four main conditions, namely, (1) MFe_2_O_4_ + H_2_O_2_ + TPA + light, (2) MFe_2_O_4_ + TPA + light, (3) MFe_2_O_4_ + H_2_O_2_ + TPA in the dark, and (4) MFe_2_O_4_ + TPA in the dark. Conditions (1) and (2) were chosen to test the hypothesis that the primary source of photogenerated hydroxyl radicals is H_2_O_2_. Conditions (3) and (4) were chosen to determine if the ferrites produce HO^•^ from H_2_O_2_ in the dark and to provide a baseline for the experiments run under illumination. Representative fluorescence spectra from these experiments are shown in Figure S16, and data are tabulated in Table S4.

Under condition (1), in the presence of H_2_O_2_ and light, all ferrites generate fluorescent products in various amounts. Under condition (2), illumination in the absence of H_2_O_2_, much less fluorescence is detected for Fe, Co, and Zn ferrites. This comparison demonstrates that these ferrites more readily generate HO^•^ radicals from the photoreduction of H_2_O_2_ than they do from reactions with water or oxygen. The exceptions to this observation are NiFe_2_O_4_, for which the already minimal amount of fluorescence remains relatively unchanged, and CuFe_2_O_4_, which shows a significant generation of fluorescence in both the presence and absence of H_2_O_2_. We note that the fluorescence spectrum observed in the absence of H_2_O_2_ for CuFe_2_O_4_ does not resemble that of hTPA (Figure S16B). Instead of a feature centered at λ ∼ 420 nm arising from hTPA, we observe a feature centered at λ ∼ 530 nm. This feature is not present in a spectrum collected for a sample of CuFe_2_O_4_ in pure water (no TPA) exposed to light (see Figure S17). We suspect that this spurious fluorescence does not arise from formation of HO^•^, but rather some other light-induced reaction involving CuFe_2_O_4_ and TPA. We also note that while conducting these control experiments, we noticed a similar longer wavelength fluorescent peak arise in solutions of MgFe_2_O_4_ in both pure water and TPA that were exposed to light (Figure S17). Since we found MgFe_2_O_4_ to generate fluorescence in the absence of TPA, we concluded that the TPA assay likely does not provide an accurate indication of the concentration of HO^•^ produced by the MgFe_2_O_4_ nanocrystals. We therefore excluded MgFe_2_O_4_ from the TPA assay analysis.

Under condition (3), in the dark with H_2_O_2_, Fe, Co, Cu, and Zn ferrites generate smaller amounts of fluorescent products than in condition (1), whereas the already minimal amount of fluorescence detected from NiFe_2_O_4_ again remains relatively unchanged. This dark activity could result from multiple phenomena, including Fenton-like processes involving the reaction of H_2_O_2_ with Fe^2+^ or other M^2+^ species at the nanocrystal surfaces. Previous reports have observed analogous “peroxidase”-like reactivity for spinel ferrite nanocrystals in the presence of H_2_O_2_ in the dark, whereby the spinel ferrites catalyze the decomposition of H_2_O_2_, which subsequently leads to the oxidation of a small molecule colorimetric indicator.^34,63−66^ One such report that examined the same set of spinel ferrite materials studied here noted that CoFe_2_O_4_, MgFe_2_O_4_, and CuFe_2_O_4_ showed the highest “peroxidase”-like reactivity in the dark.^34^ This report posits that the differences in reactivity were due to a combination of different chemical properties of the various M^2+^ ions examined and the relative concentrations of M^2+^ and Fe^3+^ ions on the surface. Our observation that CuFe_2_O_4_ generates the most fluorescence under condition (3) is consistent with this previous work; however, our CoFe_2_O_4_ nanocrystals are comparatively less reactive relative to Fe_3_O_4_ than what was observed previously. Under condition (4), the absence of both H_2_O_2_ and light, we still observe significant fluorescence from solutions containing CuFe_2_O_4_, although the mechanism by which this occurs is unclear.

In general, we observe a positive correlation between the formation of fluorescent products under illumination in the presence of TPA and H_2_O_2_ and the percentage of methyl orange degraded under illumination (Figure 7B). These data suggest that the primary pathway for photodegradation is the photo-Fenton reaction that involves the photoinduced production of HO^•^ radicals from H_2_O_2_. To further test that this is the case, we repeated the photodegradation experiments (including the dark adsorption equilibration period) in the absence of H_2_O_2_ (Figure S18). We did not observe significant photodegradation in the absence of H_2_O_2_. These data confirm the photo-Fenton pathway as the primary mechanism of photodegradation and indicate that pathways involving generation of HO^•^ or O_2_^•–^ radicals from photoinduced charge transfer to water or oxygen, respectively, or direct charge transfer between the ferrites and methyl orange upon photoexcitation of either the ferrite or methyl orange itself do not contribute significantly to the photodegradation observed in Figure 6. We note that based on literature reports of the valence band-edge potentials of these spinel ferrites, we cannot rule out generation of HOO^•^ upon transfer of photogenerated holes from the ferrite to H_2_O_2_ (see Table S5).

### Metal Ferrites’ Surface Charge Dictates Adsorption of Methyl Orange

**Figure 8:**
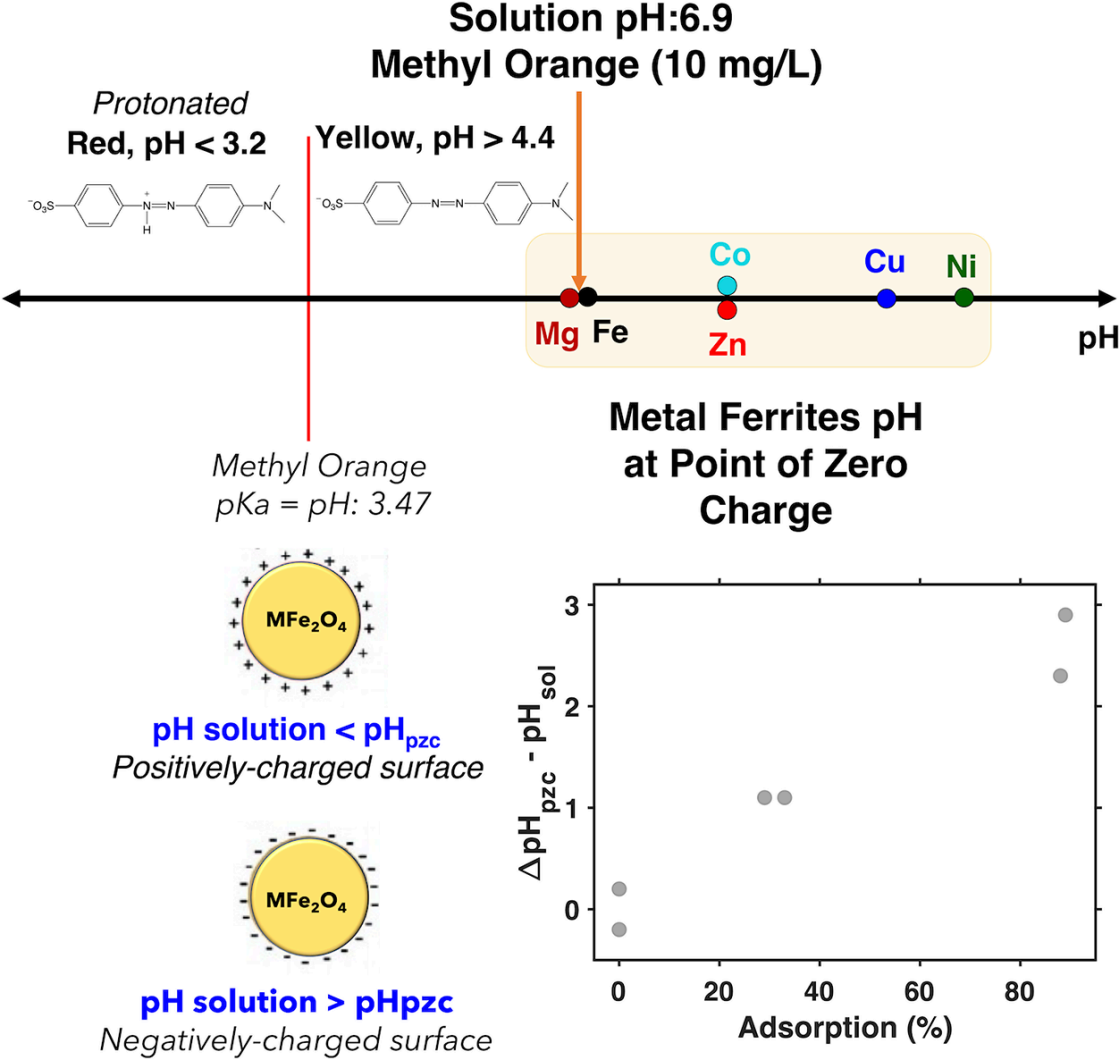
(top) Plot of a pH scale showing the relative positions of the pH_pzc_ of the metal ferrites, the pH of a 10 mg/L solution of methyl orange, and the p*K*_a_ of methyl orange. (bottom left) Schematic depicting changes in the surface charge of metal ferrites depending on how the pH of the solution compares to the pH_pzc_. If the pH of the solution is less than the pH_pzc_, then the surface of the ferrite will have a net positive surface charge, and if the pH of the solution is greater than the pH_pzc_, then the ferrite will have a net negative surface charge. (bottom right) Plot of the difference between the pH_pzc_ and solution pH (pH_sol_) versus the percent of methyl orange removed from solution in the dark.

Aiming to investigate why methyl orange molecules adsorb to some but not all ferrite surfaces in the dark, we performed pH analyses to determine the effect that surface charge could have on the observed adsorption behavior and examined the relationship between surface charge and surface oxygen speciation. Previous reports have demonstrated that the pH_pzc_ of a metal oxide semiconductor can impact its ability to remove organic dyes from solution.^67^ If the pH value of the initial solution is less than the pH_pzc_ of the metal ferrite, the ferrite surface charge is positive and it could favor adsorption of anionic compounds, whereas if the pH value of the initial dye solution is greater than the pH_pzc_ of the metal ferrite, its surface would be negatively charged and it would tend to adsorb cationic compounds.^68^ We observe that the initial pH of our methyl orange solution is neutral, ∼6.9. Since the p*K*_a_ of the azo moiety in methyl orange is 3.47,^69^ at a pH of 6.9, the azo group is predominantly unprotonated and the dye has a net negative charge due to the deprotonated sulfonate group (Figure 8). The pH_pzc_ for each ferrite can be found in Table 3 (see Figure S19 for details of how these values were determined). In the case of the ferrites that showed more adsorption under dark conditions, namely, Co, Ni, Cu, and Zn ferrites, we observe that their pH_pzc_ is higher than the pH of the methyl orange solution, suggesting a positively charged surface, which in turn can induce favorable adsorption of the anionic nonprotonated dye via electrostatic interactions (Figure 8). We note that these materials do not adsorb a neutral molecule, *p*-nitrophenol, in the dark (see Figure S20). This observation supports our hypothesis that the observed dark adsorption behavior with methyl orange is driven by Coulombic attraction.

**Table 3:** pH at Point Zero Charge of the Studied Metal Ferrites and Changes in the pH of the Methyl Orange Solution after Addition of the Catalyst^a^

metal ferrite	pH_pzc_	pH after adding the catalyst and H_2_O_2_ to the methyl orange solution
MgFe_2_O_4_	6.7	7
Fe_3_O_4_	7.1	7
CoFe_2_O_4_	8	7.4
NiFe_2_O_4_	9.8	8.3
CuFe_2_O_4_	9.2	7.6
ZnFe_2_O_4_	8	7.8

a See the Supporting Information for details of how these values were obtained.

**Figure 9:**
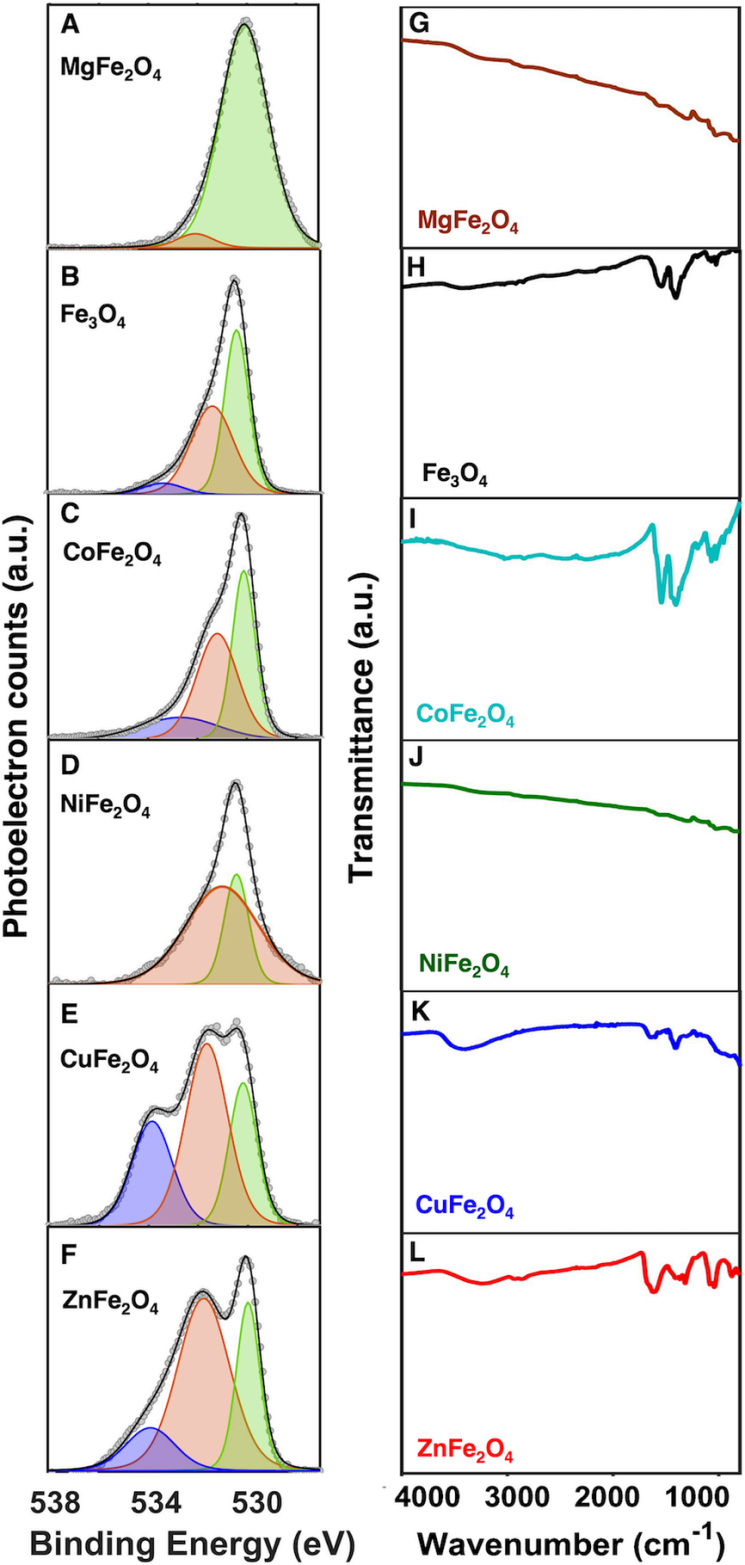
(A–F) High-resolution XPS spectra of the O 1s region displaying three main oxygen species, namely lattice oxygen (green), oxygen defects (orange), and surface-bound H_2_O (blue). (G–L) ATR-FTIR spectra of spinel metal ferrites.

To investigate the origins of the different pH_pzc_ values observed for the different ferrite materials, we examined the speciation of oxygen on the surfaces of the ferrite nanocrystals using X-ray photoelectron spectroscopy. The XPS full scan spectra of ferrites (Figure S21) confirmed the existence of all expected elements with no peaks associated with the silicon substrate observed. The high-resolution O 1s spectra for all spinel ferrites exhibit asymmetric peaks, indicating that multiple oxygen species are present at the surface (Figure 9A–F). Each spectrum was fit to two or three Gaussian components, which we have classified as O_a_, O_b_, and O_c_, centered at binding energies of 530, 531.5, and 533 eV, respectively. The relative areas of these respective peaks are tabulated in Table S5.

The O_a_ component is characteristic of O^2–^ ions of the surface lattice oxygen and possesses the lowest binding energy.^70^ It is represented by a green Gaussian fit. We assign the O_b_ component centered around 531.5 eV and represented by an orange Gaussian fit to the presence of surface oxygen defects. Several previous reports assign a similar O 1s peak observed in metal oxide materials to oxygen atoms adjacent to oxygen vacancies (V_O_);^71−73^ however, other reports indicate that this peak more likely corresponds to surface hydroxyls or chemisorbed water molecules.^74−78^ A recently reported DFT study using ZnO as a model system demonstrates that surface-bound hydroxyl species produce a binding energy that is 1.5 eV higher than that for lattice oxygen anions, consistent with the 531.5 eV binding energy observed here.^74^ Furthermore, these calculations indicate that oxygen vacancies cause only minor (≪1 eV) changes to the binding energies of adjacent oxygen atoms. Other reports point out that surface oxygen vacancies can be rapidly healed upon exposure to ambient H_2_O via dissociative adsorption to form surface hydroxyl species.^75−78^ In-situ XPS data collected of oxygen-deficient metal oxides upon deliberate introduction of water and subsequent heating confirm the assignment of the peak centered at a binding energy of 531.5 eV to surface hydroxyl species. We therefore consider surface hydroxyls to be the most likely species responsible for the O_b_ feature observed in our materials.

We assign the O_c_ peak centered at 533 eV to physisorbed water molecules,^79^ and it is represented by a blue Gaussian fit. We corroborated our assignment of the O_c_ peaks to surface-bound water molecules via FTIR measurements. Figure 9G–L features representative FTIR spectra of the metal ferrites. Fe, Co, Cu, and Zn ferrites show broad bands at 3100–3200 cm^–1^. These broad bands are attributed to the stretching and bending modes of the O–H bonds in surface-bound water molecules. The broad line width is characteristic of the impact of hydrogen bonding on the O–H stretching frequency. Hydrogen-bonding interactions could arise between the water molecules and surface oxygen atoms or between water molecules bound to adjacent surface sites. The materials that contain these broad FTIR bands are also the ferrites that exhibit the largest contribution from the O_c_ peaks in XPS. This observation is consistent with our assignment of the O_c_ peaks to surface-bound water molecules.

Overall, we find that the ferrites with significant concentrations of surface hydroxyl groups and surface-bound H_2_O molecules as measured by XPS, namely, Co, Ni, Cu, and Zn ferrite, are also the ferrites that exhibit a higher pH_pzc_ and significant dark adsorption of methyl orange (Figure 8). These data indicate that the presence of surface groups that can accept (HO^–^) or release (H_2_O, HO^–^) protons influences pH_pzc_ and, consequently, dark adsorption behavior. Finally, we note that when mixing the Ni and Zn ferrite catalysts with hydrogen peroxide and the methyl orange solution, a significant increase in the initial pH of the methyl orange solution (from pH ∼ 6.9 to 8.3 and 7.8, respectively) is observed. This increase in pH indicates an increase in the concentration of ^–^OH anions that can compete with the methyl orange dye molecules for surface sites on the nanocrystal. This competition could influence the equilibrium in the dark and may be a factor in the observed release of previously adsorbed methyl orange molecules upon the addition of H_2_O_2_ and light in these ferrites. It is also possible that photoisomerization of adsorbed methyl orange^80^ may play a role in this photorelease process. Indeed, the fundamental reasons as to why the displacement of adsorbed methyl orange molecules occurs with these ferrites upon the addition of H_2_O_2_ and light are still an open question.

### Effect of Reusability in the Removal of Methyl Orange

Ultimately, it is important to develop catalysts that enable low-cost and less labor-intensive recovery from effluent streams and possess the ability to withstand repeated usage and attrition since the recovery and reuse of the catalyst particles after water treatment continues to be the main technical barrier that impedes the commercialization of effective photocatalysts.^81^ We sought to evaluate the performance of the Fe_3_O_4_ nanocrystals for three repeated methyl orange degradation reaction cycles. After each cycle, we collected the Fe_3_O_4_ nanocrystals via centrifugation, washed them 3 times with a 2:1 mixture of water/ethanol, and dried them at 100 °C for 3 h before adding them to a fresh solution containing 10 mg/L of methyl orange and repeating the degradation reaction. Figure S22 displays a 4.0% and 3.0% loss in photodegradation efficiency after the second and third cycles, respectively, suggesting these nanocrystals could be repeatedly utilized without significant performance loss.

We also examined the reusability of a ferrite that exhibited significant adsorption of methyl orange in the dark in addition to photoinduced degradation activity, namely CuFe_2_O_4_. CuFe_2_O_4_ nanocrystals collected after a degradation reaction via centrifugation and washing as described above do not induce any removal of methyl orange in the dark but still induce degradation of >90% of the methyl orange molecules under illumination (see Figure S23). These data are consistent with our interpretation that the removal process that occurs in the dark is a surface adsorption process that fills all available surface sites for methyl orange binding but does not impede the degradation activity under illumination. Furthermore, the lack of methyl orange removal in the dark during the second round of experiments indicates that these surface binding sites remain occupied throughout the nanocrystal collection and reprocessing procedure.

## Summary and Future Outlook

We observe a positive correlation between the intensity of fluorescence detected in a fluorescent assay for HO^•^ upon illumination of mixtures of metal ferrite nanocrystals and H_2_O_2_ and the fraction of methyl orange molecules removed under illumination in the presence of these same metal ferrite nanocrystals. This observation is consistent with the proposed mechanism that photogenerated hydroxyl radicals are the primary oxidants that induce degradation of methyl orange. We also observe that organic ligands present on the surface of nanocrystals impede the photogeneration of hydroxyl radicals and that the adsorption of methyl orange to the ferrite surfaces in the dark is correlated to the ferrite’s surface charge. These observations demonstrate the importance of identifying surface structure/photodegradation function relationships to enable the development of design principles for improving the photodegradation performance of spinel ferrites. We find that Fe_3_O_4_ exhibits the highest photodegradation activity, although the origins of this superior performance are not clear from our data. We directly demonstrate that the ferrite materials are more effective at inducing photodegradation upon exposure to the filtered output (λ > 350 nm) of a Xe arc lamp than TiO_2_. Finally, Fe_3_O_4_ can be collected and easily reused without a significant decrease in its performance toward photoinduced degradation. Our observation that CuFe_2_O_4_ nanocrystals induce methyl orange removal in the dark on only the first trial is consistent with the assignment of this dark removal process to surface adsorption.

This work illustrates the complexity of photoinduced degradation processes on nanostructured semiconductor materials and reveals several open questions regarding how surface chemistry impacts the behavior of these systems. Our results indicate that the dark adsorption process is driven by Coulombic attraction between the surface of the metal ferrites and the methyl orange molecules. Our observation of different pH_pzc_ values for different MFe_2_O_4_ materials suggests that the identity of the M metal may influence the ferrites’ surface charge. However, there are other factors that may also contribute. These include the M/Fe ratio at the ferrite surface, the metal:oxygen ratio at the surface, and the coordination environment of these sites (i.e., tetrahedral versus octahedral). Spinel ferrites are known to exhibit various distributions of the metal cations among the tetrahedral and octahedral crystal sites,^41^ and this cation distribution may also play a role in determining the surface charge. Notably, all of these variables can be tuned within the same spinel ferrite composition, i.e., same identity of M, by changing synthetic conditions, such as annealing temperature,^33,82^ solvothermal reaction solvent and ligands,^83^ or applying postsynthetic surface chemical treatments with e.g. NaBH_4_.^84−87^ Further work is needed to establish the extent to which these factors impact the pH_pzc_ and, consequently, the surface charge at a given reaction pH. The mechanism of the photoinduced desorption of methyl orange observed in NiFe_2_O_4_ and ZnFe_2_O_4_ is also a mystery.

Another, more general, question that merits further investigation is the relative impact of surface structures related to oxygen (e.g., surface hydroxyl species and surface-bound water) and the composition of the metal ferrite on photodegradation performance. It is possible that differences in surface oxygen speciation may contribute to variations in reactivities observed across different reports examining nominally the same metal ferrite compositions. It is also not clear how much the surface oxygen chemistry depends on the identity of the metals present in the metal ferrite or whether it is possible to tune the surface oxygen chemistry independently of the metal ferrite composition and thereby tune the surface charge. Furthermore, we note that the photo-Fenton mechanism involves generation of Fe^2+^ ions that then subsequently reduce H_2_O_2_. This mechanism implies the localization of photogenerated charge carriers to Fe^3+^ sites at or near the surface of the ferrite. Thus, the availability and redox potential of iron sites on the surface may impact the photodegradation rate. The probability that a photogenerated electron will localize to a surface Fe^3+^ site is dependent on its mobility and lifetime. Carrier mobility in spinel ferrites occurs via small polaron hopping mechanisms and is known to depend on the identity of M, cation distribution, and the presence of defects such as oxygen vacancies.^88−91^ Lifetimes of photogenerated charge carriers almost certainly depend on these same factors. Disentangling the impacts of changes in the photophysical characteristics on the photodegradation activity of spinel ferrites from the impacts of changes in the surface structure and chemistry remains a major challenge.

Finally, the most significant practical question relevant to developing these materials as viable photodegradation agents for removal of organic pollutants is why such large ferrite concentrations are required to observe the photodegradation of methyl orange and whether the need for large concentrations is general across multiple pollutant molecules. Precise calculation of the stoichiometry of these reactions is complicated by the heterogeneous nature of the nanomaterials and the lack of knowledge of the structure of the active surface species. However, all the studies listed in Table 1 clearly use an excess of the photoactive material. On the other hand, several studies, including this one, have shown that the nanocrystals can be recovered and reused for multiple cycles with negligible losses in activity (see Figure S23). This reusability suggests that the active component of the nanocrystals is not consumed or diminished by the reaction, which, along with acceleration of the reaction rate, is a defining feature of a catalyst.

We suspect that this large concentration requirement is due to two factors. First, the tendency of ligandless nanocrystals to aggregate in solution makes a significant fraction of the nanocrystal surface inaccessible to substrates such as H_2_O_2_. Second, in practical applications of these systems, the target organic pollutants will be found in trace amounts. In fact, the United States Environmental Protection Agency’s thresholds for maximum allowable concentrations of regulated organic molecules in drinking water are well below 10 ppm.^92^ It therefore makes sense to study how well a photoactive material can remove molecules that are present at very low concentrations. Most of the data documented in Table 1 as well as the original data we report here correspond to initial pollutant concentrations of 10 ppm or less. We suspect that running the photodegradation reactions at substoichiometric concentrations of the nanocrystals when the concentration of the pollutant is already so dilute severely limits light absorption by the nanocrystals and significantly slows the degradation process, particularly when the target pollutant is a dye like methyl orange that strongly absorbs visible light and further impedes absorption by the photoactive material. It therefore makes practical sense to run these reactions at superstoichiometric concentrations of metal ferrite to obtain reasonable kinetics. We note that the need to use superstoichiometric amounts of metal ferrite to observe significant degradation does not necessarily preclude these materials from being used in real applications since many photodegradation reactor designs involve flowing tainted water over a film or through a membrane containing immobilized photocatalyst,^93,94^ where the local concentration of pollutant in contact with the photocatalyst is lower than the concentration of photoactive material.

## Supporting Information Available

Characterization of metal ferrite nanocrystals by powder X-ray diffraction, transmission electron microscopy, energy-dispersive X-ray spectroscopy, X-ray photoelectron spectroscopy, diffuse reflectance spectroscopy, and ^1^H NMR spectroscopy; UV–vis spectra for the methyl orange and nitrophenol removal experiments, fluorescence spectra for the TPA assay experiments; calculations for mol % MFe_2_O_4_ as well as relative surface iron concentrations; Xe arc lamp emission spectra; details of the determination of pH_pzc_, and kinetics of degradation of methyl orange over repeated uses of the Fe_3_O_4_ and CuFe_2_O_4_ (PDF)
