# Supplementary material for: Surface Adsorption and Photoinduced Degradation: A Study of Spinel Ferrite Nanomaterials for Removal of a Model Organic Pollutant from Water
Source: Chem Mater. 2024 Apr 24;36(9):3981–98. doi: 10.1021/acs.chemmater.3c01986 (PMC11099926; doi:10.1021/acs.chemmater.3c01986)
Supplement: Supplementary file 1 — cm3c01986_si_001.pdf [file cm3c01986_si_001.pdf]

Supporting Information for

Surface Adsorption and Photoinduced Degradation: A Study of Spinel Ferrite Nanomaterials for Removal of a Model Organic Pollutant from Water

Karla R. Sanchez-Lievanos, Tong Sun, Elise A. Gendrich, and Kathryn E. Knowles*

Department of Chemistry, University of Rochester, Rochester, NY 14627, United States

*Corresponding author: Email address: kknowles@ur.rochester.edu

Table of Contents

Table S1. Calculation of surface metal sites available in $ZnFe_2O_4$ for the determination of mol % based on fraction of surface metals. S3

Scheme S1. Photoinduced degradation experimental setup. S4

Figure S1. Transmission spectrum of the water filter used in the photodegradation measurements. S4

Figure S2. Size distribution of $NiFe_2O_4$ NCs with different surface ligands determined from analysis of transmission electron microscopy images. S5

Figure S3. Powder XRD patterns of surface-functionalized $NiFe_2O_4$ NCs. S5

Figure S4. Paramagnetic ^{1}H NMR of ligand exchanged $NiFe_2O_4$ NCs solution in deuterated DMSO S6

Figure S5. Representative ^{1}H NMR spectra in D_2O of the successful ligand exchange S7

Atomic Absorption Calculations S7

Table S2. Calculation to determine the volume to use for a 25 mol % $NiFe_2O_4$ in a 20 mL solution of 10 mg/L methyl orange. S8

Figure S7. UV-Vis spectra of photodegradation experiments run with ligand-echanged $NiFe_2O_4$ NCs and single-source precursor molecules S9

Figure S8. Xe Arc Lamp emission spectra S10

Figure S9. Photographs of methyl orange solution with and without metal ferrite NCs S10

Fluorescence Assay for Production of Hydroxyl Radicals S11

Figure S10. Representative fluorescence spectra collected during fluorescent TPA assay of ligand-exchanged $NiFe_2O_4$ nanocrystals subjected to H_2O_2 and illumination S12

Figure S11. EDS Spectra of all metal ferrites S13

Figure S12. Kinetics of degradation of the methyl orange dye solution in three main control scenarios in the absence of MFe_2O_4 .... S13

Table S3. Calculation for mol % MFe_2O_4 used in photodegradation experiments S14

Figure S13. UV-Vis spectra of photodegradation experiments of methyl orange S14

Diffuse Reflectance Measurements. S15

Figure S14. Diffuse reflectance spectra of ligandless metal ferrite powders S15

Figure S15. Characterization of the transmission of the Xe arc lamp through representative suspensions of metal ferrite powders used for photocatalysis. S16

Figure S16. Representative fluorescence spectra collected during fluorescent TPA assay of ligandless MFe_2O_4 particles subjected to various illumination and H_2O_2 conditions S17

Table S4. Integrated fluorescence intensities of solutions of MFe_2O_4 and TPA collected under different conditions and percentage photocatalytic degradation.. S17

Figure S17. Fluorescence spectra collected of $MgFe_2O_4$ in the presence and absence of TPA. S18

Figure S18. Results of photodegradation control experiments conducted in the absence of H_2O_2. .... S19

Table S5. Literature Values of Valence Band-Edge Potentials S20

Method for determining the pH at the point of zero charge (pH_{pzc}).. S20

Figure S19. Plots of ΔpH versus pH_i used to calculate the pH_{pzc}... S21

Figure S20. Dark adsorption control experiment conducted with p-nitrophenol..................................... S22

Figure S21. XPS survey spectra of all metal ferrites S23

Table S6. Speciation of Surface Oxygen in Various Metal Ferrites Measured by XPS S23

Figure S22. Kinetics of degradation of methyl orange over three repeated uses of Fe_3O_4. .... S24

Figure S23. Kinetics of degradation of methyl orange over the first repeated use of $CuFe_2O_4$. S24

Table S1. Calculation of surface metal sites available in $ZnFe_2O_4$ for the determination of mol % based on fraction of surface metals.

lattice parameter	Diameter (nm)	radius (nm)	Zn (per unit cell)	Fe (per unit cell)	volume of single particle (nm^3)	volume of unit cell (nm^3)	# Unit cells per particle
0.842	210	105	8	16	4.85×10^6	0.597	**8.12×10^6**
**Ionic diameter of Zn^{2+} (nm)**	**Ionic diameter of Fe^{3+} (nm)**	**Average ionic diameter (nm)**	**Radius nanoparticle - average ionic diameter**	**Core diameter (nm)**	**core volume (nm^3)**		**# unit cells in core**
0.148	0.128	0.138	104.9	209.7	4.83×10^6		**8.09×10^6**

Unit cells on surface = $8.12 \times 10^6 - 8.09 \times 10^6 = 3.2 \times 10^4$

Total surface Zn: $8*3.2 \times 10^4 = 2.56 \times 10^5$

Total surface Fe: $16* 3.2 \times 10^4 = 5.12 \times 10^5$

Total metal ions available @ surface: $3.2 \times 10^4 + 5.12 \times 10^5 = 7.68 \times 10^5$

Total metal ions available = (8.12×10^6 unit cells*16 Fe) + (8.12×10^6 unit cells*8 Zn) = 1.95×10^8

surface metals/ total metals: = $7.68 \times 10^5/1.95 \times 10^8 = 0.004$

Concentration of oxide	Total molarity of oxide	molarity of surface metal ions	Molarity of pollutant (terramycin – 0.04g/L)	mol surface/mol pollutant	% NC based on fraction of surface metals
0.6g/L ($ZnFe_2O_4$)	0.0025	1×10^{-5}	$8.7\text{x}10^{-5}$	0.11	11

Scheme S1. Photoinduced degradation experimental setup.

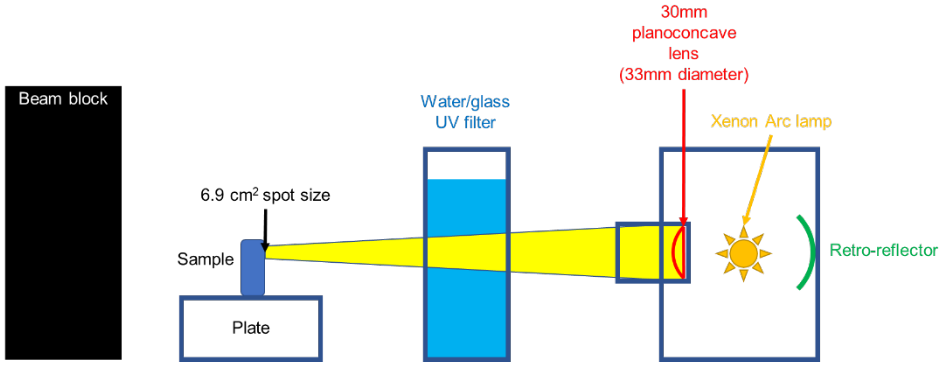

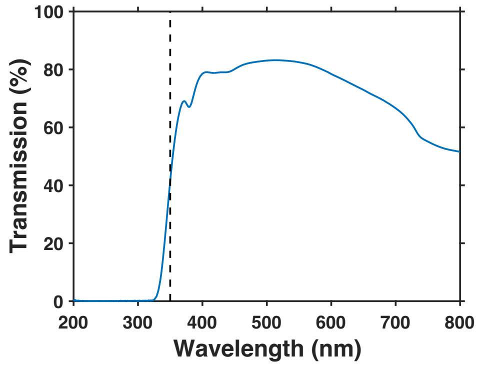

Figure S1. Transmission spectrum of the water filter used in the photodegradation measurements. The vertical dashed line indicates the cutoff wavelength of 350 nm, which we define to be the wavelength at which the transmission is 50% of its maximum.

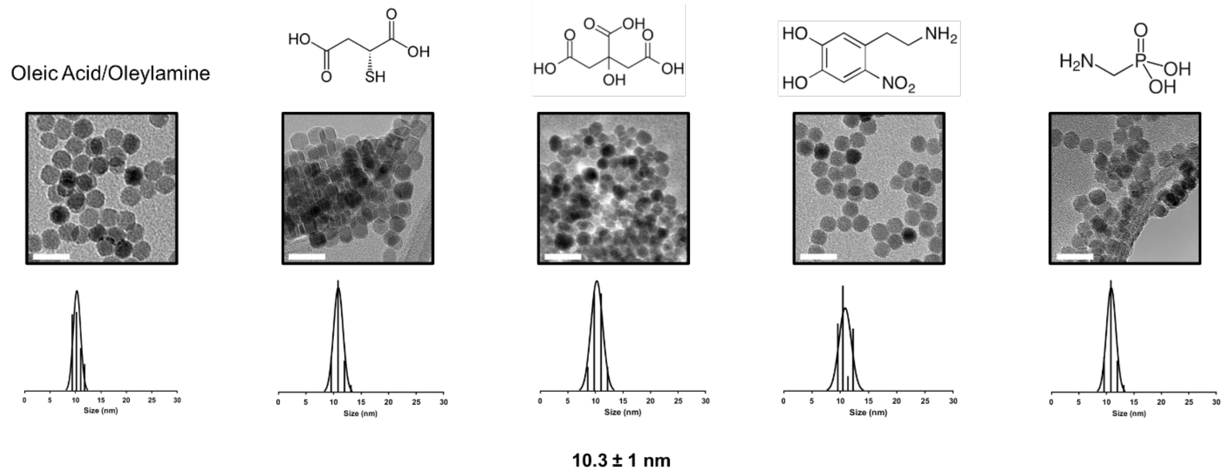

Figure S2. Size distribution of $NiFe_2O_4$ NCs with different surface ligands determined from analysis of transmission electron microscopy images. From left to right: oleic acid, mercaptosuccinic acid, citric acid, nitrodopamine and aminomethyl phosphonic acid. Scale bars: 20 nm.

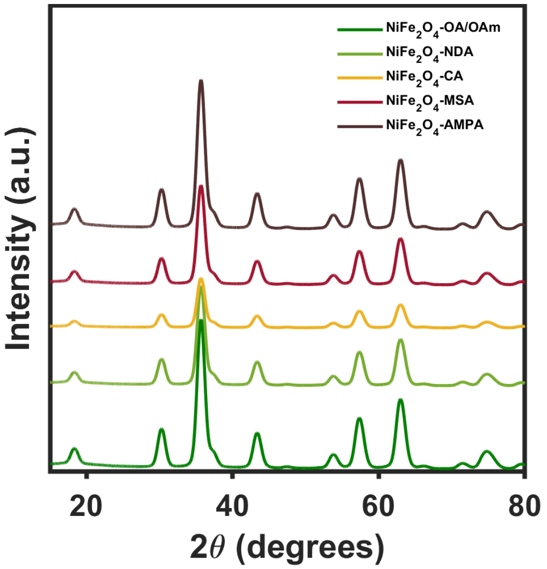

Figure S3. Powder XRD patterns of surface-functionalized $NiFe_2O_4$ NCs.

'

To distinguish broadening due to the paramagnetic nature of our nanocrystals from broadening due to the steric consequences of being bound to a nanocrystal surface, we added excess ligands to nanocrystal dispersions. If the broadening is primarily due to surface binding effects, then we would expect addition of excess ligands to produce sharp peaks indicative of unbound species. We found that after adding 1 mmol of CA to a solution of $NiFe_2O_4$-CA in deuterated DMSO, a slightly sharper peak from CA started to appear; however, after adding 1 mmol more CA to the solution, the peak broadened out again, which indicates the contributions of broadening due to paramagnetism (Figure S4).

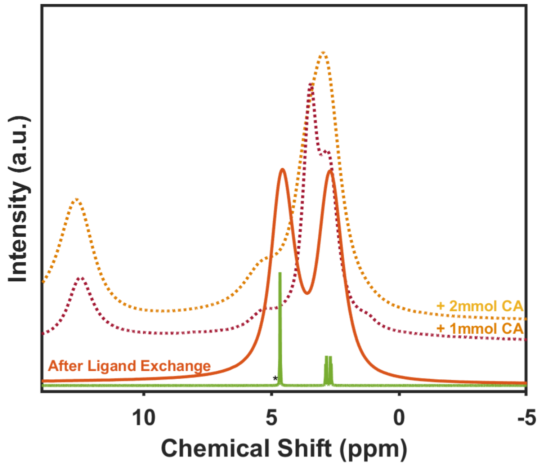

Figure S4. Paramagnetic ^{1}H NMR of ligand exchanged $NiFe_2O_4$ NCs solution in deuterated DMSO (*). The addition of 2 mmol of citric acid (CA) does not show any signals of unbound ligands.

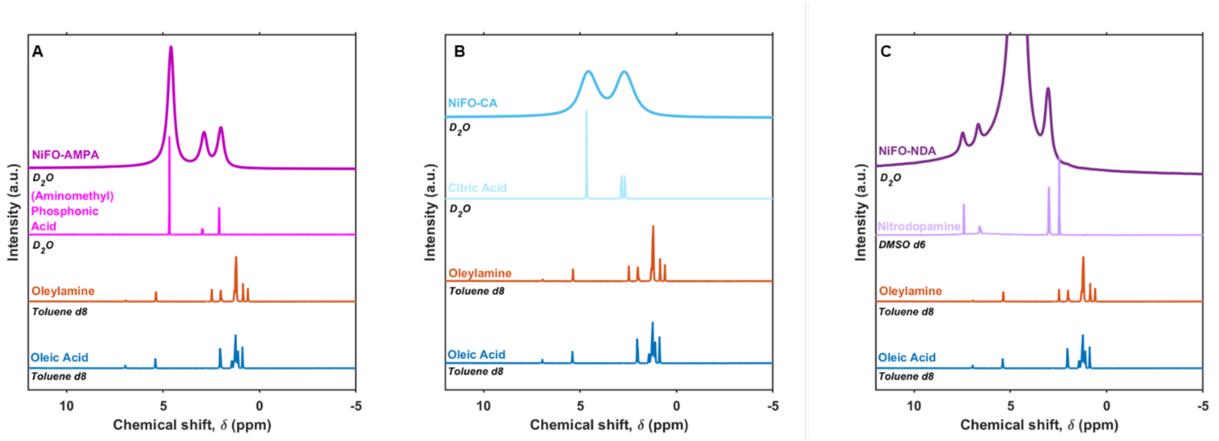

Figure S5. Representative ^{1}H NMR spectra in D_2O of the successful ligand exchange of OA/Oam ligands in $NiFe_2O_4$ for aminomethyl phosphoric acid, citric acid, and nitrodopamine. The peak at $\delta \sim 5$ ppm in the D_2O spectra corresponds to trace H_2O.

Atomic Absorption Calculations to determine the content of iron in colloidal dispersions of $NiFe_2O_4$ NCs and calculate the μL of stock solution needed for a 25 mol % nanocrystal concentration.

Calibration curve

PPM	ABS
0	0.0018
0.8764	0.094
2.0817	0.1836
3.821	0.3129
4.8742	0.3912
8.2183	0.6398
9.5406	0.7381
12.357	0.9469

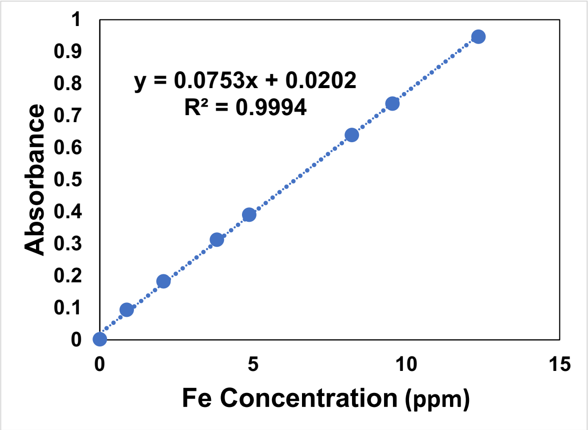

Figure S6. Plot of Fe atomic absorbance versus Fe concentration used to calibrate the atomic absorption measurements.

Table S2. Calculation to determine the volume to use for a 25 mol % $NiFe_2O_4$ in a 20 mL solution of 10 mg/L methyl orange.

**NiFO**	**PPM (Fe)**	**AA ABS**	**TOTAL mL AA Sample**	**mL Stock solution**	**Stock solution Concentration (ppm Fe)**	**Bulk Molarity of Fe (mol/L)**	**mol % NiFO**	**moles of Fe ($x10^{-7}$)**	**V (µL)**
NiFO NDA	1.33	0.11	14.2119	0.1375	137	0.0025	25	3.06	**125**
NiFO AMPA	4.43	0.35	13.9943	0.2455	253	0.0045	25	3.06	**67**
NiFO MSA	5.44	0.43	14.2021	0.172	449	0.0080	25	3.06	**38**
NiFO CA	5.97	0.47	14.1662	0.1513	559	0.0100	25	3.06	**31**
NiFO SSP TFA	11.58	0.89	14.3556	0.1451	1146	0.0205	25	3.06	**15**
NiFO SSP Oleate	0.93	0.09	14.2566	0.0007	18880	0.3381	25	3.06	**1**

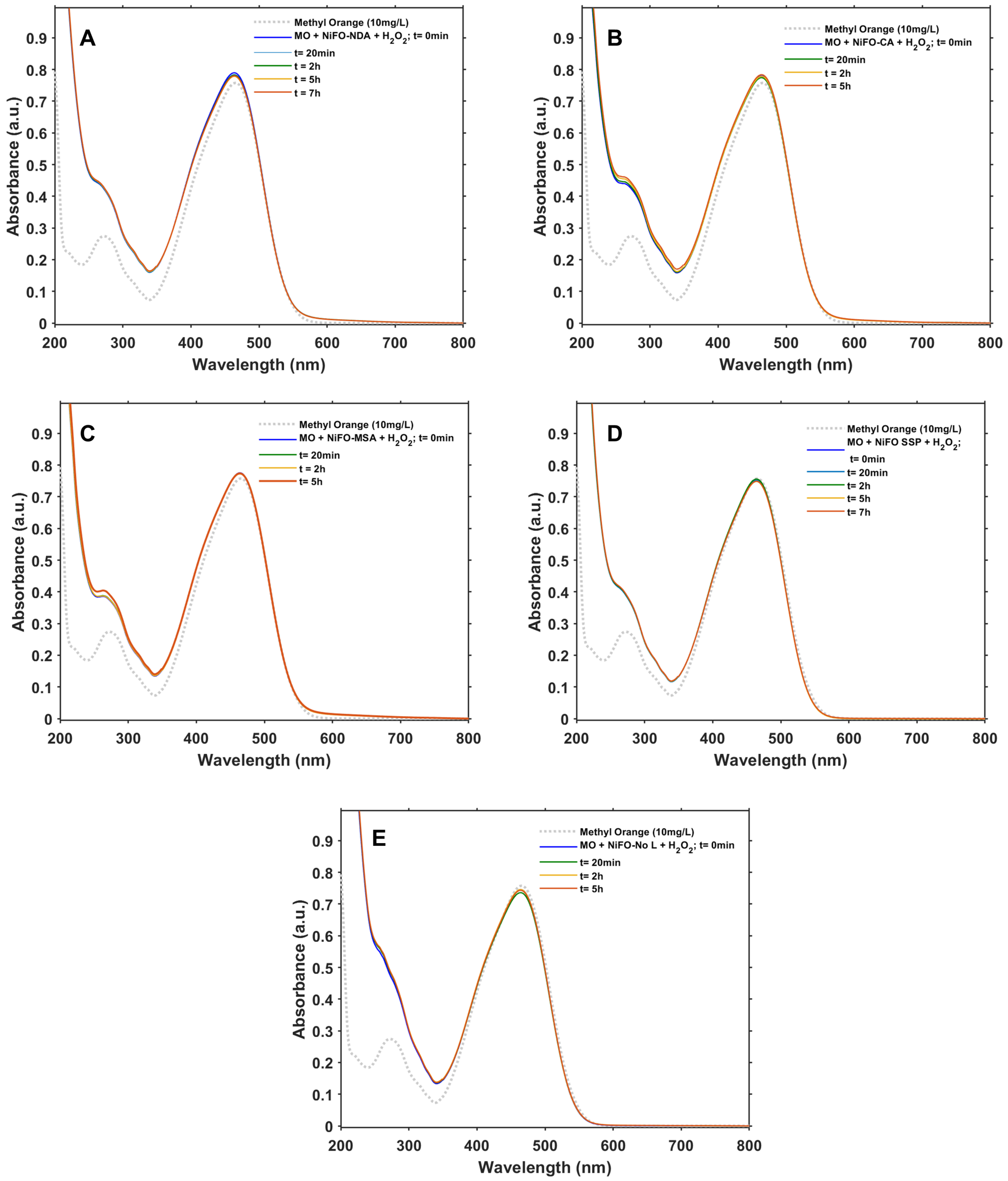

Figure S7. UV-Vis spectra of solutions of methyl orange (10 mg/L), 6.4 mM H_2O_2 and 250 mol % of nanocrystals or single source precursors exposed to illumination for various amounts of time. A) $NiFe_2O_4$ functionalized with nitrodopamine (NDA), B) $NiFe_2O_4$ functionalized with citric acid (CA), C) $NiFe_2O_4$ functionalized with mercaptosuccinic acid (MSA), D) $NiFe_2(\mu_3\text{-}O)(\mu_2\text{-}O_2CCF_3)_6(H_2O)_6$, E) $NiFe_2O_4$ with no ligands.

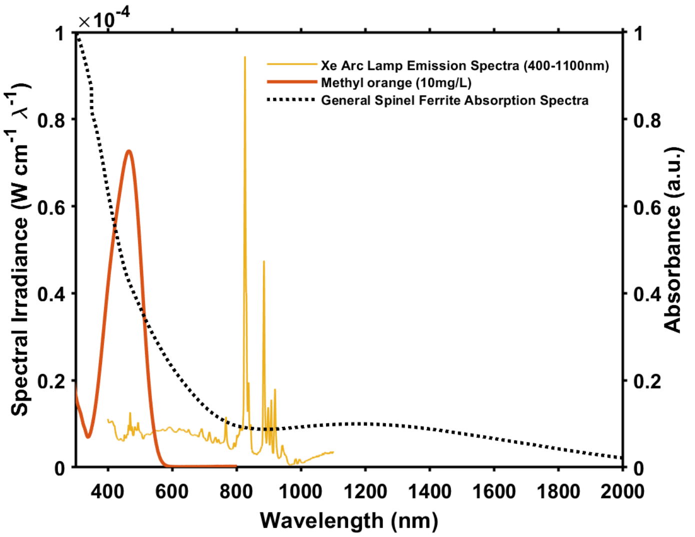

Figure S8. Xe Arc Lamp emission spectra (yellow line), methyl orange absorption spectrum (orange line, 10 mg/L) and spectrum of a solution of $NiFe_2O_4$ nanocrystals corresponding to 25 mol% ferrite (1.53×10^{-7} M $NiFe_2O_4$). We note that the detector used to measure the lamp spectrum is only sensitive to visible and some near-infrared light (400 mm, < λ < 1100 nm), so we are unable to characterize the UV portion of the lamp spectrum.

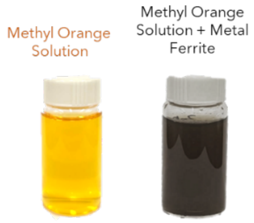

Figure S9. Representative photographs of methyl orange solution before and after adding the metal ferrite nanocrystals.

Fluorescence Assay for Production of Hydroxyl Radicals

TPA is a commonly used chemical probe sensitive to hydroxyl radicals and with which other reactive oxygen species (H_2O_2, $HO^{2\bullet}$, $O_2^{\bullet-}$) are reported not to interfere.[1,2] In solution, hydroxyl radicals react with TPA to form the fluorescent molecule 2-hydroxyterephthalic acid (hTPA) as the primary product (Figure 4 inset), although a recent analysis indicates that other products that fluoresce at the same wavelength as hTPA may also form.[1] With oxygen present, hTPA yield has been reported to be up to 35%.[3] This methodology has been previously utilized in a number of investigations into various systems for the production of hydroxyl radicals including metal oxides,[4-6] metal organic frameworks,[7] and other nanoscale materials.[8] For most of the nanocrystal samples examined here, upon mixing with TPA, H_2O_2, and illumination, we observe a fluorescence spectrum consistent with formation of hTPA, however for some samples we observe an additional fluorescent product. We therefore use the total integrated fluorescence intensity as a semi-quantitative handle to assess the relative concentrations of reactive hydroxyl radicals generated under various conditions. Figure S10 shows representative fluorescence spectra obtained from solutions containing $NiFe_2O_4$ nanocrystal samples with various surface ligands and TPA subjected to illumination in the presence H_2O_2. The dashed dark gray line in each spectrum corresponds to the fluorescence spectrum of a solution containing hTPA for reference. These data demonstrate that some nanocrystal samples that show significant fluorescence, likely contain fluorescent species in addition to hTPA. The observation of multiple species with overlapping fluorescence spectra upon reaction of TPA with $HO^\bullet$ has been reported previously in the literature.[1] Due to the difficulty in quantifying these mixtures, we use the total integrated fluorescence intensity as a semi-quantitative measure to assess the relative ability of these nanocrystal systems to generate $HO^\bullet$ under various conditions. To ensure that experiments conducted for the set of $NiFe_2O_4$

nanocrystals with various surface ligands are comparable to those conducted for the various spinel ferrites synthesized without ligands, in figures 4 and 7A in the main text we normalize the data to a common sample: ligandless $NiFe_2O_4$ nanocrystals exposed to H_2O_2 under illumination for 5 h. We suspect that the sharp feature observed at ~410 nm in some spectra is due to a fluorescent impurity in a cuvette since it is also observed in spectra collected of pure water under the same excitation conditions.

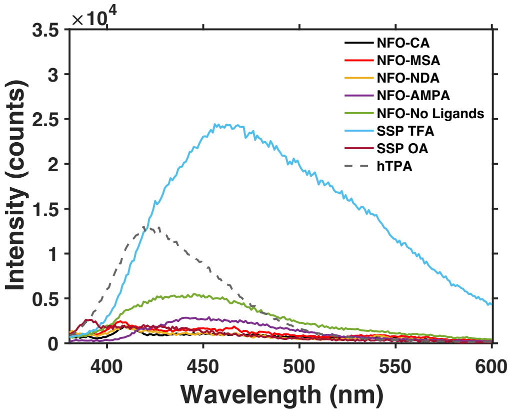

Figure S10. Representative fluorescence spectra collected of solutions containing TPA, 6.4 mM H_2O_2, and colloidal $NiFe_2O_4$ nanocrystals with various surface ligands or single-source Ni-O-Fe_2 precursor molecules. The dashed gray line represents a fluorescence spectrum collected for a solution of hTPA in Nanopure water.

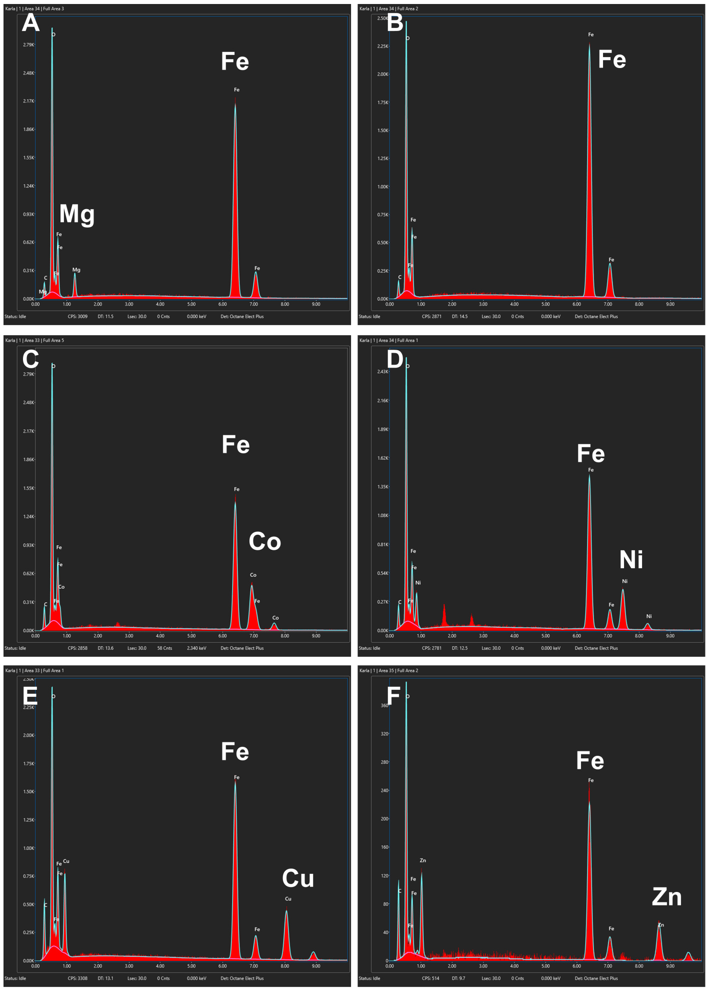

Figure S11. EDS Spectra of all metal ferrites showcasing the presence of all expected elements in the structure. (**A**) $MgFe_2O_4$, (**B**) Fe_3O_4, (**C**) $CoFe_2O_4$, (**D**) $NiFe_2O_4$, (**E**) $CuFe_2O_4$, and (**F**) $ZnFe_2O_4$

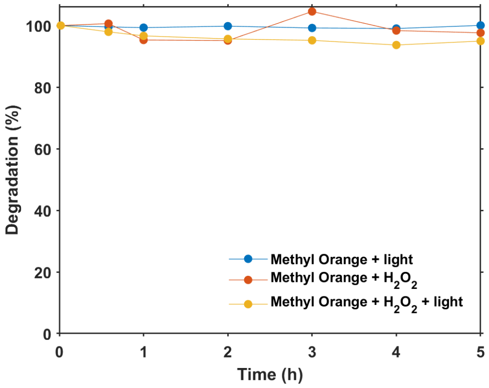

Figure S12. Kinetics of degradation of the methyl orange dye solution in three main control scenarios in the absence of MFe_2O_4

Table S3. Calculation for mol % MFe_2O_4 used in photodegradation experiments

Ferrite	Molar Mass	cat load (g/L)	Mol/L	Methyl Orange (mol/L)	mol MFe_2O_4/mol methyl orange	%
$MgFe_2O_4$	199.99	0.5	0.0025	3.06E-05	81.84	8184
Fe_3O_4	231.53	0.5	0.0022	3.06E-05	70.69	7069
$CoFe_2O_4$	234.62	0.5	0.0021	3.06E-05	69.76	6976
$NiFe_2O_4$	234.38	0.5	0.0021	3.06E-05	69.83	6983
$CuFe_2O_4$	239.23	0.5	0.0021	3.06E-05	68.41	6841
$ZnFe_2O_4$	241.08	0.5	0.0021	3.06E-05	67.89	6789

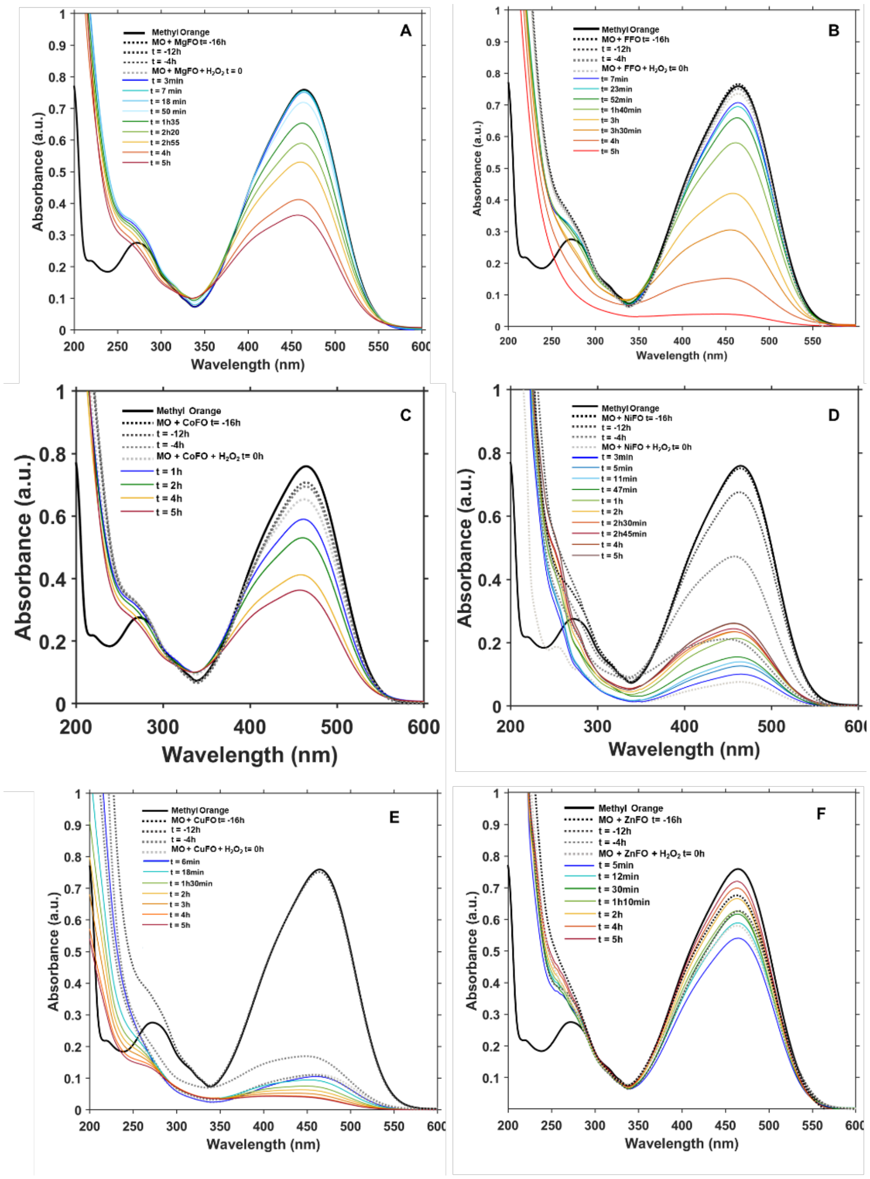

Figure S13. UV-Vis spectra of photodegradation experiments of methyl orange (10 mg/L) with H_2O_2 (6.4 mM) and spinel metal ferrites (0.5g/L) (**a**) $MgFe_2O_4$, (**b**) Fe_3O_4, (**c**) $CoFe_2O_4$, (**d**) $NiFe_2O_4$, (**e**) $CuFe_2O_4$, and (**f**) $ZnFe_2O_4$.

Diffuse Reflectance Measurements.

Diffuse reflectance spectra were collected of films of the MFe_2O_4 powders dropcast onto glass microscope slides. These spectra were collected using a home-built setup comprised of an incandescent tungsten lamp as a light source and a fiber-coupled Ocean Optics array detector. The diffuse reflectance was collected and focused onto the optical fiber using an air-spaced doublet collimator (ThorLabs F810SMA-543, 24 mm diameter) oriented perpendicular to the path of the input tungsten lamp. A microscope slide wrapped in Teflon tape was used as the 100% reflectance standard. The angle of the samples was adjusted to exclude specular reflectance from the measurement. The Kubelka-Munk transformation (eq. S1) was performed by the Ocean Optics software. In equation S1, $R_{MFe_2O_4}$ is the intensity of the reflected light collected from the ferrite sample and R_0 is the intensity of the reflected light collected from the 100% reflectance standard.

$$A = \frac{(1-R)^2}{2R}, R = \frac{R_{MFe_2O_4}}{R_0} \quad \text{(S1)}$$

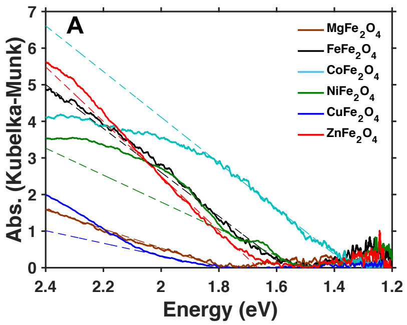

B

Material	Absorption Onset (eV)
$MgFe_2O_4$	1.79
Fe_3O_4	1.57
$CoFe_2O_4$	1.34
$NiFe_2O_4$	1.51
$CuFe_2O_4$	1.81
$ZnFe_2O_4$	1.67

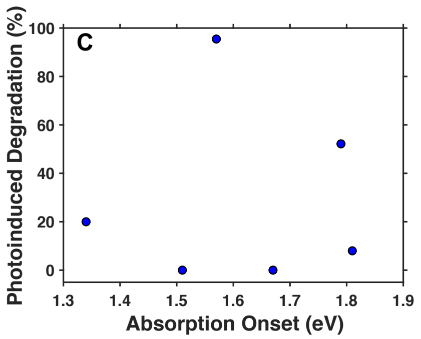

Figure S14. A) Diffuse reflectance spectra of films of ligandless metal ferrite powders on glass microscope slides. The dashed lines represent the linear fits used to estimate the abosrption onsets. **B**) Table listing the absorption onsets estimated from the x-intercepts of the linear fits shown in part A. **C**) Plot of the percent of methyl orange removed from solution via photodegradation in the presence of MFe_2O_4 versus the absorption onset of the MFe_2O_4 material.

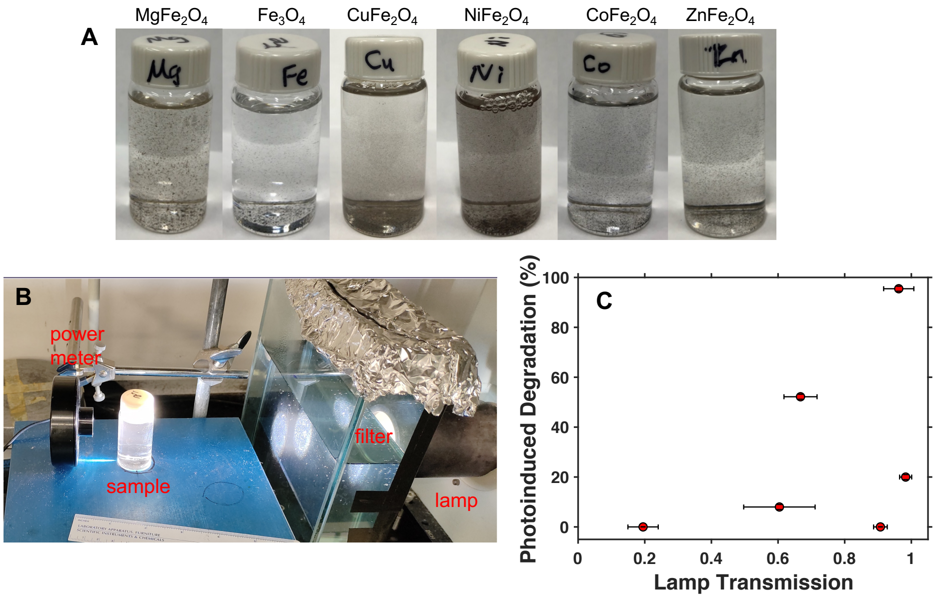

Figure S15. A) Photographs of representative suspensions of metal ferrite powders used for photocatalysis. **B**) Photograph of experimental setup used to measure the total transmission of the Xe arc lamp through these suspensions. Transmission is defined as the power measured through the ferrite suspension divided by the power measured through a vial of pure water. **C**) Plot of the percent of methyl orange removed from solution via photodegradation in the presence of MFe_2O_4 versus the transmission of the lamp through the ferrite suspension.

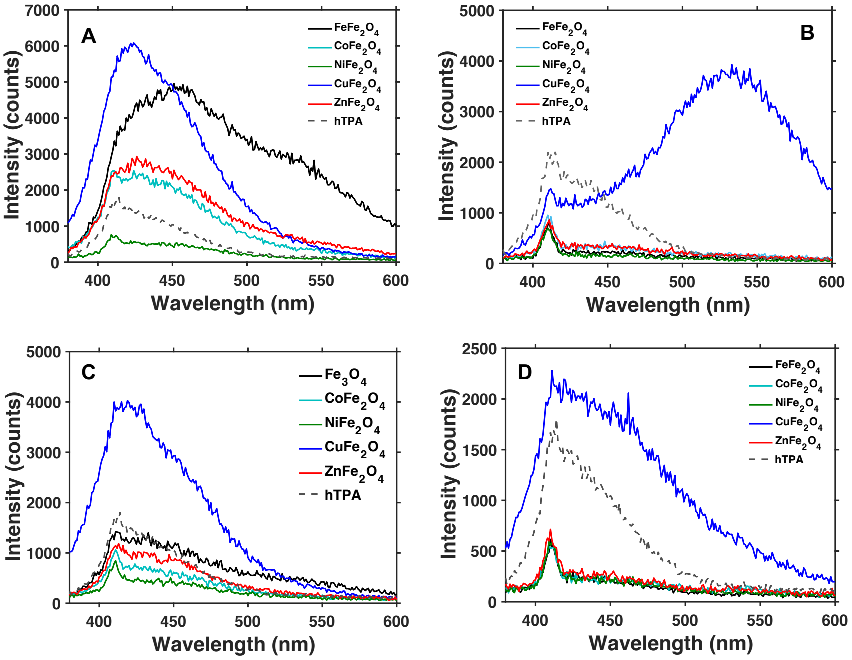

Figure S16. Representative fluorescence spectra collected of (**A,C**) solutions containing various ligandless MFe_2O_4 nanocrystals, TPA, 6.4 mM H_2O_2, and (**B,D**) solutions containing various ligandless MFe_2O_4 nanocrystals and TPA only. Solutions shown in parts (**A**) and (**B**) were subjected to illumination for 5 hours prior to fluorescence collection, whereas solutions shown in parts (**C**) and (**D**) were stirred in the dark for 5 hours and 16 hours, respectively, prior to fluorescence collection. The dashed gray lines in each plot represent fluorescence spectra collected for a solution of hTPA in Nanopure water. All spectra were collected with an excitation wavelength of 360 nm, slit widths of 0.75 mm and an integration time of 150 ms.

Table S4. Integrated fluorescence intensities of solutions of MFe_2O_4 and TPA collected under different conditions and percentage photocatalytic degradation[a]

Ferrite	MFe_2O_4, TPA, H_2O_2 under light ($\times 10^5$)[b]	MFe_2O_4, TPA, under light ($\times 10^5$)[b]	MFe_2O_4, TPA, H_2O_2 in dark ($\times 10^5$)[b]	MFe_2O_4, TPA, in dark ($\times 10^5$)[c]	Photocatalytic Degradation (%)
Fe_3O_4	6.43 ± 0.13	0.32 ± 0.04	1.46 ± 0.01	0.317 ± 0.002	96 ± 0.9
$CoFe_2O_4$	2.27 ± 0.01	0.54 ± 0.09	0.74 ± 0.01	0.368 ± 0.004	20 ± 0.7
$NiFe_2O_4$	0.590 ± 0.005	0.6 ± 0.5	0.53 ± 0.02	0.336 ± 0.005	0
$CuFe_2O_4$	5.13 ± 0.01	4 ± 1	3.42 ± 0.03	2.42 ± 0.01	8 ± 0.6
$ZnFe_2O_4$	2.79 ± 0.02	0.4 ± 0.3	0.98 ± 0.01	0.409 ± 0.002	0

[a]Uncertainties represent the standard deviation of three separate trials. [b]Experiments run for 5 hours. [c]Experiments run for 16 hours.

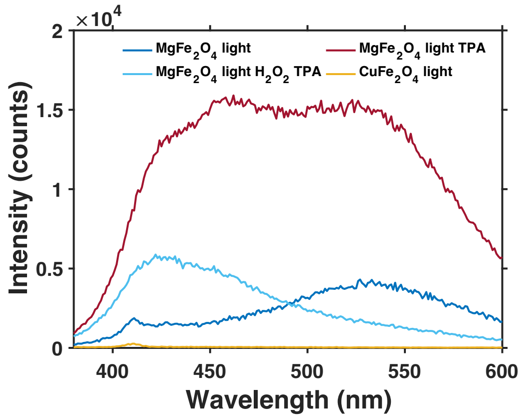

Figure S17. Fluorescence spectra collected of the following solutions after exposure to the filtered output of the Xe arc lamp and one pass through a syringe filter: $MgFe_2O_4$ (dark blue), $MgFe_2O_4$ with 7 mM TPA and 6.4 mM H_2O_2 (light blue), $MgFe_2O_4$ and 7 mM of TPA (dark red), $CuFe_2O_4$ (yellow). The fluorescent feature centered at $\lambda \sim 530$ nm appears upon exposure of the $MgFe_2O_4$ sample to light in the absence of TPA or H_2O_2. A similar feature appears in the spectrum collected for $CuFe_2O_4$ exposed to light and TPA (Figure S16B) but is absent from a solution containing $CuFe_2O_4$ alone. The appearance of this feature in the absence of H_2O_2 or TPA complicates the use of the fluorescent assay to quantify the formation of $HO^{\bullet}$ radicals in samples containing $MgFe_2O_4$. We therefore exclude $MgFe_2O_4$ from the fluorescence assay analysis.

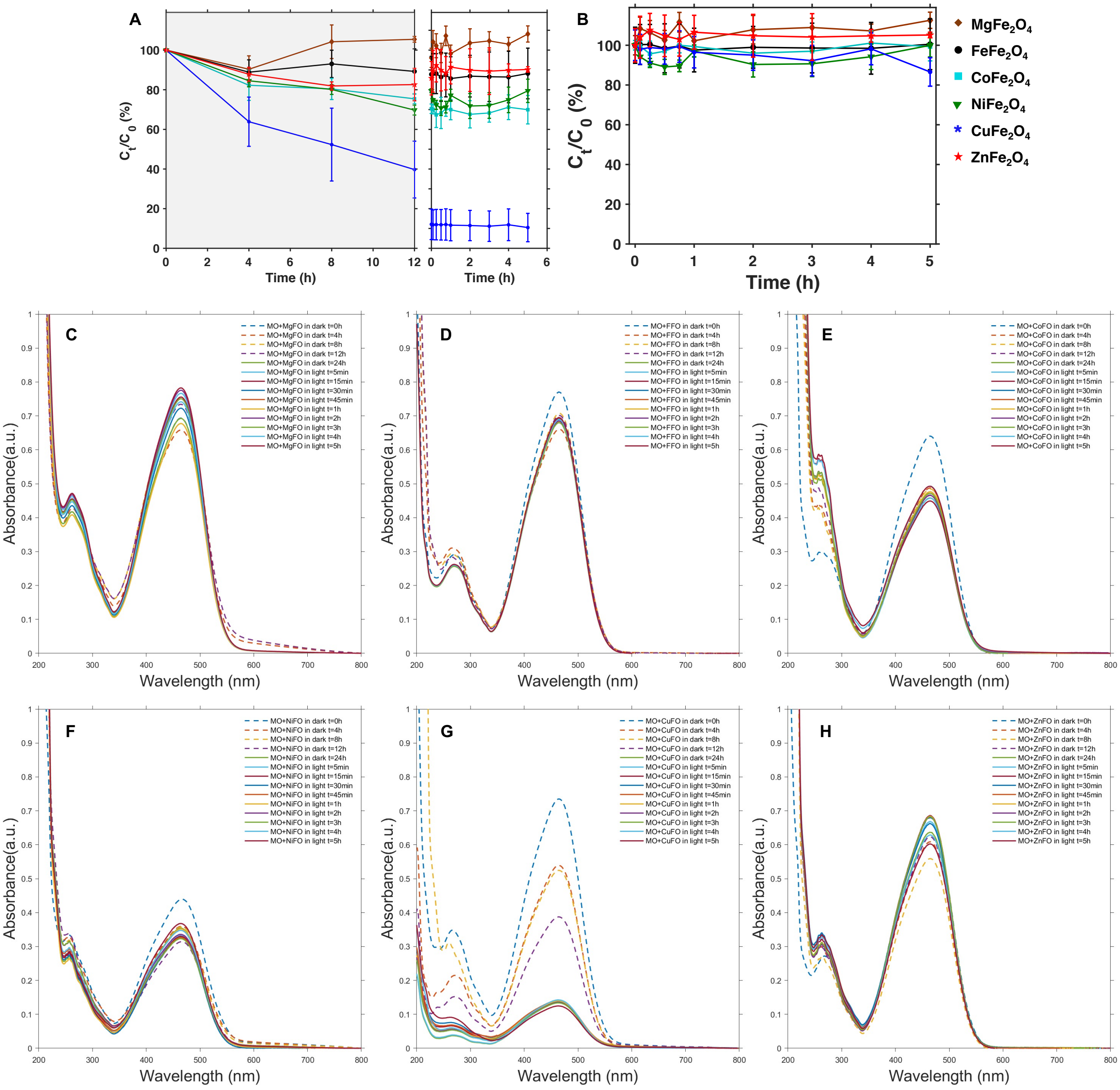

Figure S18. A) Plot of the change in concentration as measured by UV-Vis spectroscopy of a 10 mg/L methyl orange solution versus time in the dark (left) and under illumination (right) in the presence of 0.5 g/L MFe_2O_4 and absence of H_2O_2. **B)** Plot of the change in methyl orange concentration under illumination in the absence of H_2O_2 normalized to t = 0 h. **C-H**) Representative UV-Vis spectra collected for mixtures containing 10 mg/L of methyl orange and 0.5 g/L of C) $MgFe_2O_4$, D) Fe_3O_4, E) $CoFe_2O_4$, F) $NiFe_2O_4$, G) $CuFe_2O_4$, and H) $ZnFe_2O_4$ after various time points in the dark and under illumination.

Table S5. Literature Values of Valence Band-Edge Potentials

**Ferrite**	**E_{VB} (V vs. NHE at pH 7)[a]**	**References**
$MgFe_2O_4$	1.38-1.73	[9,10]
Fe_3O_4	1.51-1.85	[11,12]
$CoFe_2O_4$	1.07-1.55	[13,14]
$NiFe_2O_4$	1.12-1.3	[15,16]
$CuFe_2O_4$	0.62-0.74	[17-19]
$ZnFe_2O_4$	1.69-2.09	[20,21]
**Redox Reaction**	**E (vs. NHE at pH 7)[a]**	**References**
$HO_2^{\bullet} + H^+ + e^-/H_2O_2$	1.03	[22]
$HO^{\bullet} + H^+ + e^-/H_2O$	2.15	[22]

[a]When needed, values reported vs. NHE at different pH values were converted to NHE at pH 7 using the equation V(NHE at pH 7) = V(NHE) + (pH-7)*0.059.

Method for determining the pH at the point of zero charge (pH_{pzc})

We determined the pH at the point of zero charge (pH_{pzc}) of metal ferrite nanocrystals using the powder addition method.[23,24] We prepared 10 solutions of 0.1 M $NaNO_3$ and tuned their pH to values of 3, 4, 5, 6, 7, 8, 9, 10, 11, and 12 using a Fisher Scientific Accumet AE150 Benchtop pH meter to measure the pH. These values are used as pH_i. The metal ferrite oxide powder (10 mg) was added to 20 mL of each solution. The solutions were mixed at 150 rpm on an orbital shaker for 24 hours, and then passed through 0.2 mm syringe filter. The pH of the final filtered solutions (pH_f) was measured using the pH meter. Figure S19 plots change in pH ($\Delta pH = pH_i - pH_f$). The pH_{pzc} is the value of pH_i at which $\Delta pH = 0$.

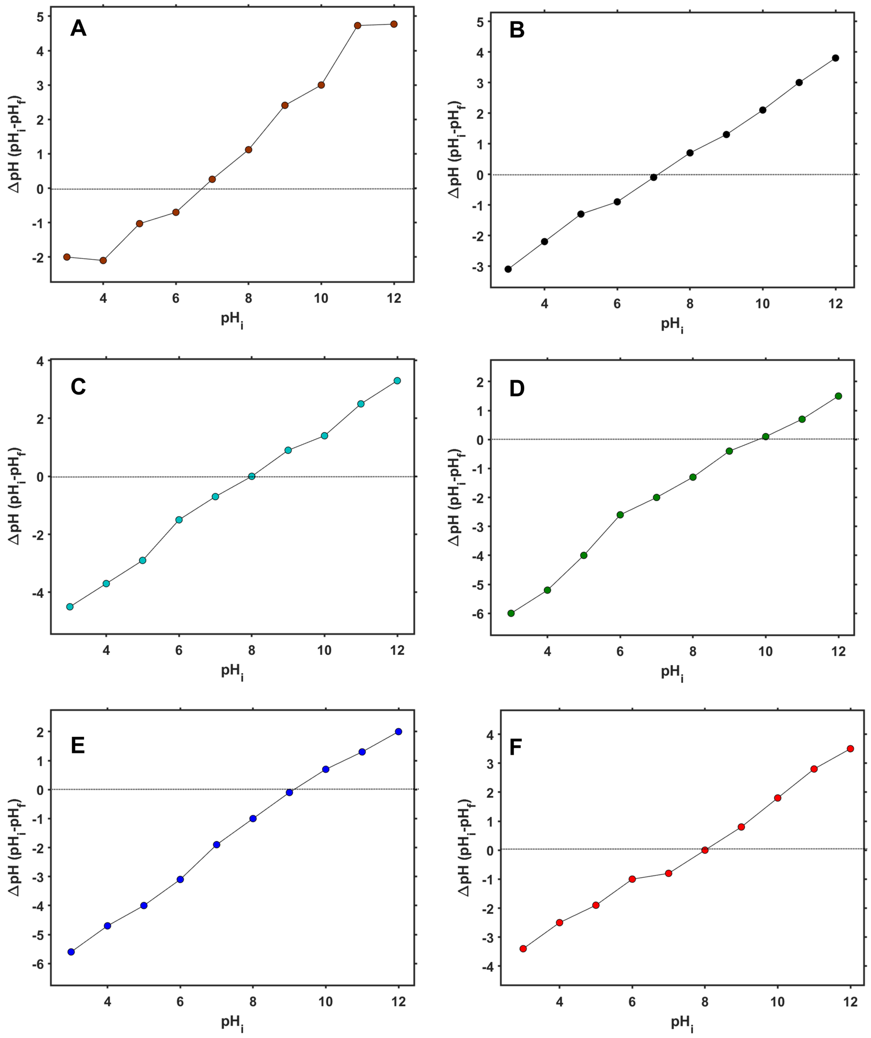

Figure S19. Plots of ΔpH versus pH_i used to calculate the pH_{pzc} of (**A**) $MgFe_2O_4$, (**B**) Fe_3O_4, (**C**) $CoFe_2O_4$, (**D**) $NiFe_2O_4$, (**D**) $CuFe_2O_4$, (**F**) $ZnFe_2O_4$.

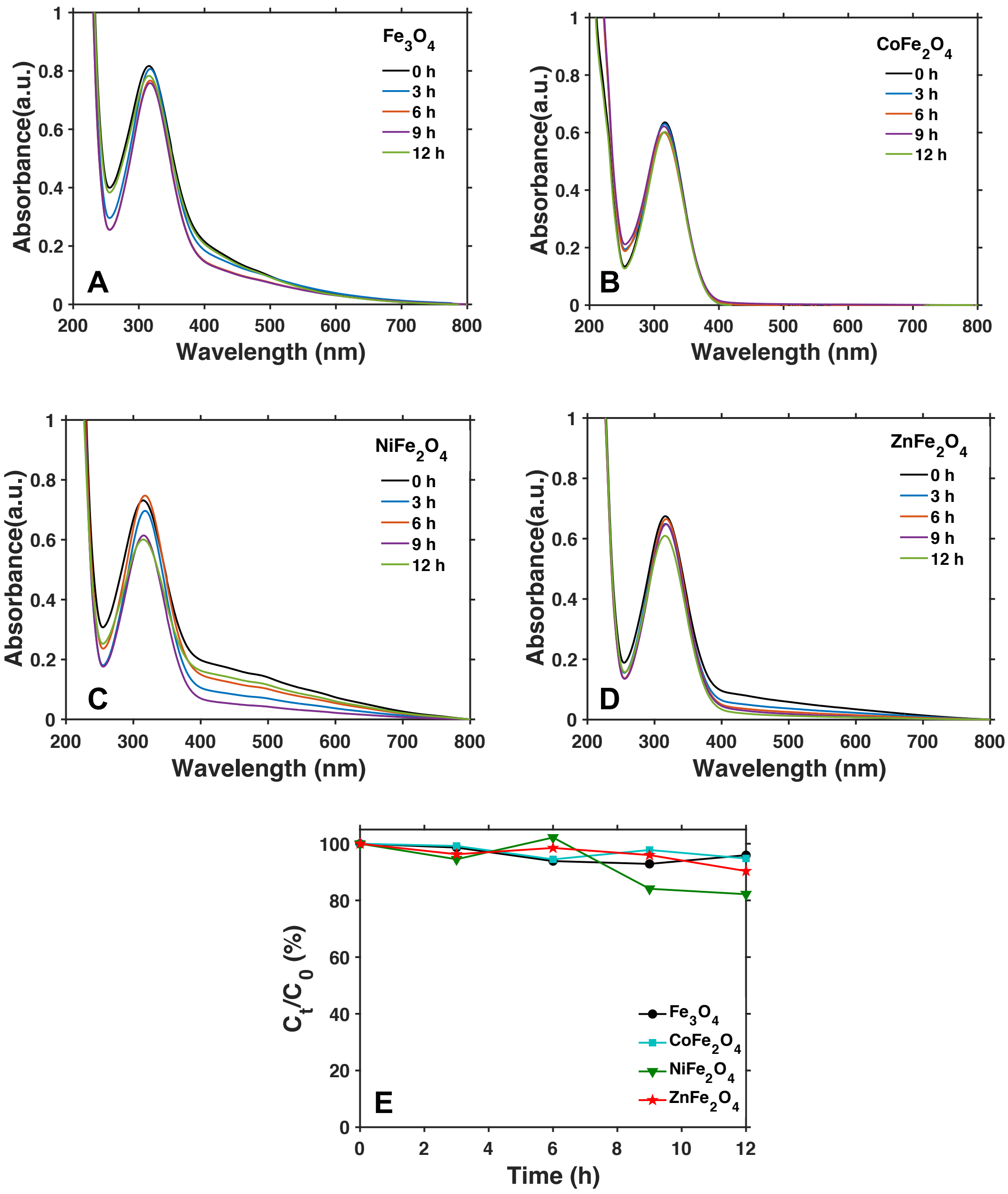

Figure S20. A-D) UV-Vis absorption spectra of filtered solutions of p-nitrophenol (pH 5) collected at various time intervals after sitting in the presence of various metal ferrites in the dark. **E**) Plot of the ratio of the absorption at $\lambda = 315$ nm at time t versus the absorption measured at t = 0 for the various solutions of nitrophenol and metal ferrites. This ratio is used to assess the change in concentration of nitrophenol in the solution and we observe very little change over the course of 12 hours.

The binding energies obtained in the XPS analysis are corrected for specimen charging by referencing C 1s at 284.8 eV. The high-resolution XPS spectra of individual elements was deconvoluted using CasaXPS software.

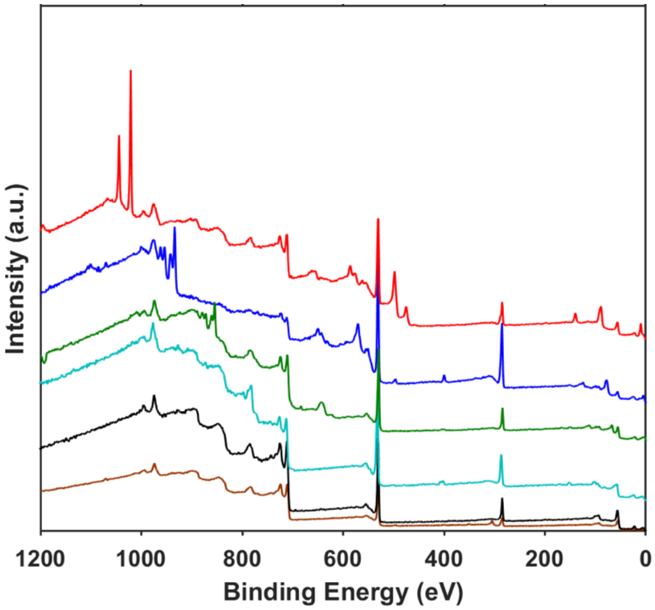

Figure S21. XPS survey spectra of all metal ferrites, from bottom to top: $MgFe_2O_4$ (brown spectrum), Fe_3O_4 (black spectrum), $CoFe_2O_4$ (light blue spectrum), $NiFe_2O_4$ (green spectrum), $CuFe_2O_4$ (dark blue spectrum), and $ZnFe_2O_4$ (red spectrum).

Table S6. Speciation of Surface Oxygen in Various Metal Ferrites Measured by XPS

Material	Lattice Oxygen (O_a)	Surface Hydroxides (O_b)	Surface-Bound H_2O (O_c)
$MgFe_2O_4$	95	5	-
Fe_3O_4	46	48	6
$CoFe_2O_4$	39	44	17
$NiFe_2O_4$	27	73	-
$CuFe_2O_4$	25	48	27
$ZnFe_2O_4$	26	61	13

.

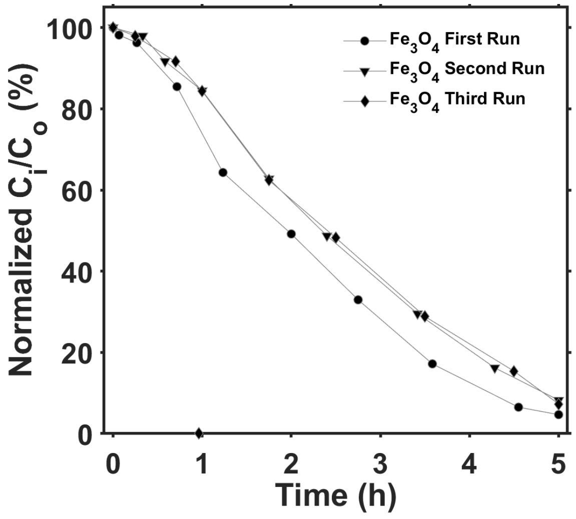

Figure S22. Kinetics of degradation of methyl orange over three repeated uses of Fe_3O_4.

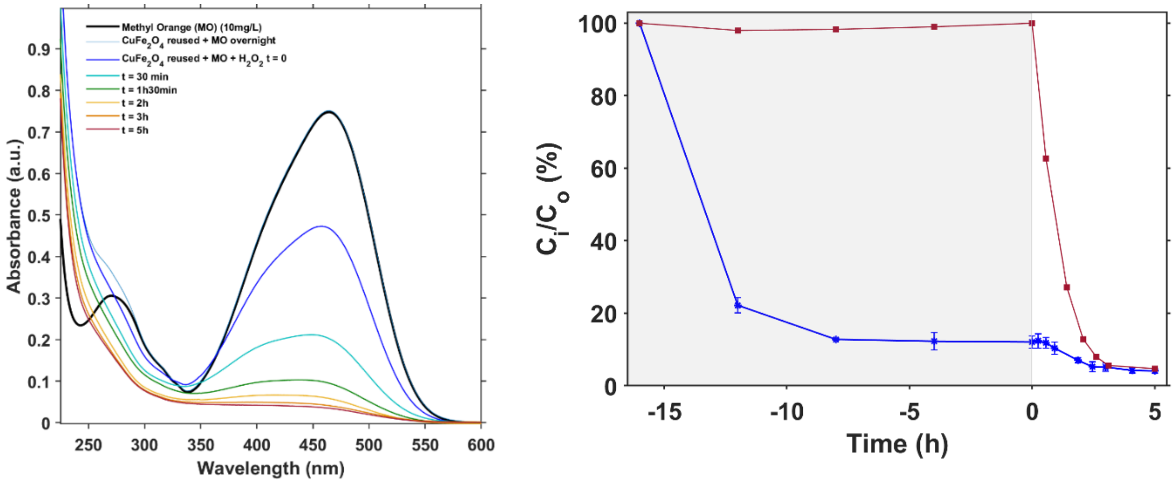

Figure S23. Kinetics of degradation of methyl orange over the first repeated use of $CuFe_2O_4$.

References

1. Tampieri, F.; Ginebra, M.-P.; Canal, C. Quantification of Plasma-Produced Hydroxyl Radicals in Solution and their Dependence on the pH. *Anal. Chem.* **2021**, *93*, 3666-3670.
2. Gonzalez, D. H.; Kuang, X. M.; Scott, J. A.; Rocha, G. O.; Paulson, S. E. Terephthalate Probe for Hydroxyl Radicals: Yield of 2-Hydroxyterephthalic Acid and Transition Metal Interference. *Anal. Lett.* **2018**, *51*, 2488-2497.
3. Fang, X.; Mark, G.; von Sonntag, C. OH radical formation by ultrasound in aqueous solutions Part I: the chemistry underlying the terephthalate dosimeter. *Ultrason. Sonochem.* **1996**, *3*, 57-63.
4. Sultana, K. A.; Islam, M. T.; Silva, J. A.; Turley, R. S.; Hernandez-Viezcas, J. A.; Gardea-Torresdey, J. L.; Noveron, J. C. Sustainable synthesis of zinc oxide nanoparticles for photocatalytic degradation of organic pollutant and generation of hydroxyl radical. *J. Mol. Liq.* **2020**, *307*, 112931.
5. Li, Y.; Li, Y.; Xu, X.; Ding, C.; Chen, N.; Ding, H.; Lu, A. Structural disorder controlled oxygen vacancy and photocatalytic activity of spinel-type minerals: A case study of $ZnFe_2O_4$. *Chem. Geol.* **2019**, *504*, 276-287.
6. Gupta, N. K.; Ghaffari, Y.; Kim, S.; Bae, J.; Kim, K. S.; Saifuddin, M. Photocatalytic Degradation of Organic Pollutants over MFe_2O_4 (M = Co, Ni, Cu, Zn) Nanoparticles at Neutral pH. *Sci. Rep.* **2020**, *10*, 4942.
7. Jing, H.-P.; Wang, C.-C.; Zhang, Y.-W.; Wang, P.; Li, R. Photocatalytic degradation of methylene blue in ZIF-8. *RSC Adv.* **2014**, *4*, 54454-54462.
8. Li, H.; Liu, R.; Lian, S.; Liu, Y.; Huang, H.; Kang, Z. Near-infrared light controlled photocatalytic activity of carbon quantum dots for highly selective oxidation reaction. *Nanoscale* **2013**, *5*, 3289-3297.
9. Kim, H. G.; Borse, P. H.; Jang, J. S.; Jeong, E. D.; Jung, O.-S.; Suh, Y. J.; Lee, J. S. Fabrication of $CaFe_2O_4/MgFe_2O_4$ bulk heterojunction for enhanced visible light photocatalysis. *Chem. Commun.* **2009**, 10.1039/B911805E, 5889-5891.
10. Jia, J.; Du, X.; Zhang, Q.; Liu, E.; Fan, J. Z-scheme $MgFe_2O_4/Bi_2MoO_6$ heterojunction photocatalyst with enhanced visible light photocatalytic activity for malachite green removal. *Appl. Surf. Sci.* **2019**, *492*, 527-539.
11. Kumar, U.; Sinha, I. Visible light photo-Fenton degradation of p-nitrophenol on $Ag/Fe_3O_4/WO_3$ nanocomposites: Experimental and molecular dynamics investigations. *J. Environ. Chem. Eng.* **2023**, *11*, 111280.
12. Xue, J.; Zhang, N.; Shen, Q.; Li, Q.; Liu, X.; Jia, H.; Guan, R. In-situ construction of photoanode with Fe_2O_3/Fe_3O_4 heterojunction nanotube array to facilitate charge separation for efficient water splitting. *J. Alloys Compd.* **2022**, *918*, 165787.
13. Kumar, A.; Kumar, A.; Sharma, G.; Al-Muhtaseb, A. a. H.; Naushad, M.; Ghfar, A. A.; Guo, C.; Stadler, F. J. Biochar-templated $g\text{-}C_3N_4/Bi_2O_2CO_3/CoFe_2O_4$ nano-assembly for visible and solar assisted photo-degradation of paraquat, nitrophenol reduction and CO_2 conversion. *Chem. Eng. J.* **2018**, *339*, 393-410.
14. Bellamkonda, S.; Chakma, C.; Guru, S.; Neppolian, B.; Rao, G. R. Rational design of plasmonic $Ag@CoFe_2O_4/g\text{-}C_3N_4$ p-n heterojunction photocatalysts for efficient overall water splitting. *Int. J. Hydrogen Energy* **2022**, *47*, 18708-18724.
15. Koutavarapu, R.; Tamtam, M. R.; Lee, S.-G.; Rao, M. C.; Lee, D.-Y.; Shim, J. Synthesis of 2D $NiFe_2O_4$ nanoplates/2D Bi_2WO_6 nanoflakes heterostructure: An enhanced Z-scheme charge transfer and separation for visible-light-driven photocatalytic degradation of toxic pollutants. *J. Environ. Chem. Eng.* **2021**, *9*, 105893.
16. Vadla, S. S.; Guru, S.; Parida, T.; John, S.; Roy, S. C.; Rao, G. R. Electrodeposited $NiFe_2O4/Cu_2O$ heterostructure thin films with enhanced photocurrent generation. *J. Photochem. Photobiol.* **2023**, *15*, 100181.
17. Bera, S.; Ghosh, S.; Maiyalagan, T.; Basu, R. N. Band Edge Engineering of $BiOX/CuFe_2O_4$ Heterostructures for Efficient Water Splitting. *ACS Appl. Energy Mater.* **2022**, *5*, 3821-3833.

18. Das, S.; Patnaik, S.; Parida, K. Dynamic charge transfer through Fermi level equilibration in the p-$CuFe_2O_4$/n-NiAl LDH interface towards photocatalytic application. *Catal. Sci. Technol.* **2020**, *10*, 6285-6298.
19. Park, S.; Baek, J. H.; Zhang, L.; Lee, J. M.; Stone, K. H.; Cho, I. S.; Guo, J.; Jung, H. S.; Zheng, X. Rapid Flame-Annealed $CuFe_2O_4$ as Efficient Photocathode for Photoelectrochemical Hydrogen Production. *ACS Sust. Chem. & Eng.* **2019**, *7*, 5867-5874.
20. McDonald, K. J.; Choi, K.-S. Synthesis and Photoelectrochemical Properties of Fe_2O_3/$ZnFe_2O_4$ Composite Photoanodes for Use in Solar Water Oxidation. *Chem. Mater.* **2011**, *23*, 4863-4869.
21. Miao, C.; Ji, S.; Xu, G.; Liu, G.; Zhang, L.; Ye, C. Micro-Nano-Structured Fe_2O_3:Ti/$ZnFe_2O_4$ Heterojunction Films for Water Oxidation. *ACS Appl. Mater. Interfaces* **2012**, *4*, 4428-4433.
22. Harris, D. C. *Quantitative Chemical Analysis*; 8th ed.; W. H. Freeman and Company: New York, NY, 2010.
23. Mustafa, S.; Dilara, B.; Nargis, K.; Naeem, A.; Shahida, P. Surface properties of the mixed oxides of iron and silica. *Colloids Surf., A* **2002**, *205*, 273-282.
24. Cristiano, E.; Hu, Y.-J.; Sigfried, M.; Kaplan, D.; Nitsche, H. A Comparison of Point of Zero Charge Measurement Methodology. *Clays Clay Miner.* **2011**, *59*, 107-115.
